# Chiral Drug Analysis in Forensic Chemistry: An Overview

**DOI:** 10.3390/molecules23020262

**Published:** 2018-01-28

**Authors:** Cláudia Ribeiro, Cristiana Santos, Valter Gonçalves, Ana Ramos, Carlos Afonso, Maria Elizabeth Tiritan

**Affiliations:** 1 Institute of Research and Advanced Training in Health Sciences and Technologies, Cooperativa de Ensino Superior Politécnico e Universitário (CESPU), Rua Central de Gandra, 1317, 4585-116 Gandra PRD, Portugal; claudia.ribeiro@iucs.cespu.pt (C.R.); cristiana.s@live.com.pt (C.S.); 2 Interdisciplinary Centre of Marine and Environmental Research (CIIMAR/CIMAR), University of Porto, Edifício do Terminal de Cruzeiros do Porto de Leixões, Av. General Norton de Matos s/n, 4050-208 Matosinhos, Portugal; cafonso@ff.up.pt; 3 Laboratory of Organic and Pharmaceutical Chemistry, Department of Chemical Sciences, Faculty of Pharmacy, University of Porto , Rua de Jorge Viterbo Ferreira, 228, 4050-313 Porto, Portugal; valter.cma@gmail.com; 4 Institute of Science and Innovation in Mechanical and Industrial Engineering (INEGI), Faculty of Engineering of the University of Porto, Rua Dr. Roberto Frias, 400, 4200-465 Porto, Portugal; shortinha.sa@gmail.com

**Keywords:** chiral drugs, forensic chemistry, enantiomers, pharmaceuticals, illicit drugs

## Abstract

Many substances of forensic interest are chiral and available either as racemates or pure enantiomers. Application of chiral analysis in biological samples can be useful for the determination of legal or illicit drugs consumption or interpretation of unexpected toxicological effects. Chiral substances can also be found in environmental samples and revealed to be useful for determination of community drug usage (sewage epidemiology), identification of illicit drug manufacturing locations, illegal discharge of sewage and in environmental risk assessment. Thus, the purpose of this paper is to provide an overview of the application of chiral analysis in biological and environmental samples and their relevance in the forensic field. Most frequently analytical methods used to quantify the enantiomers are liquid and gas chromatography using both indirect, with enantiomerically pure derivatizing reagents, and direct methods recurring to chiral stationary phases.

## 1. Introduction

**Figure 1:**
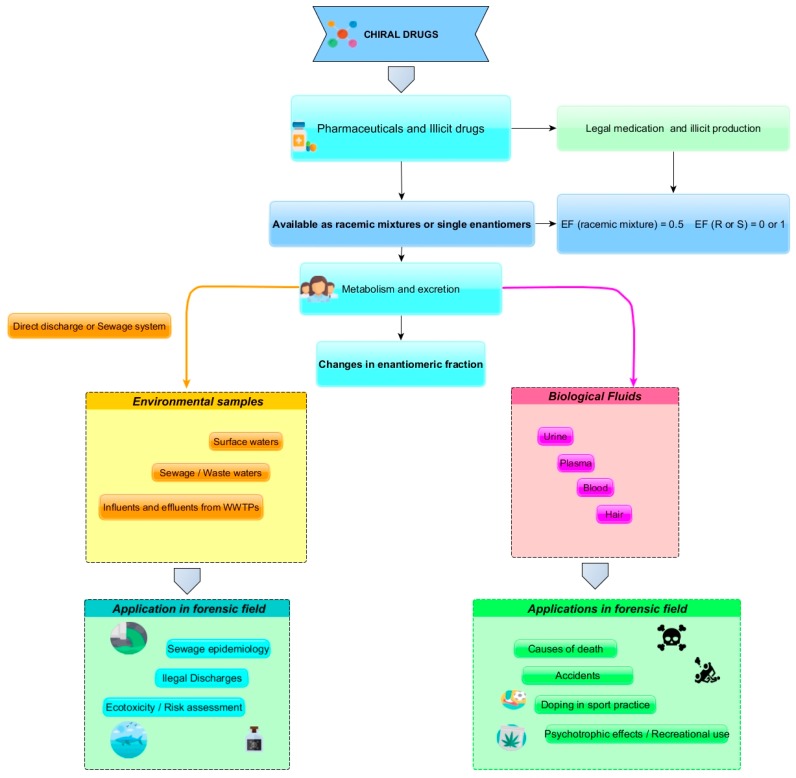
Application of chiral drug analysis in forensic chemistry.

Chiral compounds are asymmetric three dimensional molecules with one or more stereogenic centers or asymmetry originated by planes or axis that gives two non-superimposable mirror images molecules, called enantiomers [1]. In an achiral environment, a pair of enantiomers shares similar physical and chemical properties, however, in a chiral environment such as living organisms, enantiomers may exhibit different biological activities and/or toxicity due to enantioselective interactions [2,3,4]. Separation of enantiomers has gained relevance in forensic chemistry and has been applied in the analysis of biological fluids, environmental samples and in the control of illicit drug preparations [5,6,7,8,9]. Figure 1 summarizes the applications of chiral analysis in forensic chemistry.

Substances of forensic interest include pharmaceuticals and various classes of illicit drugs misused to improve sports performance or due to their psychotropic effects. The consumption of these substances can cause toxicological effects and/or increased risk of death [10,11,12,13]. These substances can be consumed by medical prescription or illicit practice and are available either as a racemates, or as a single enantiomer. Data from chiral analysis in both biological samples and illicit drugs preparations can be important in the control of manufacturing, consumption of illicit drugs or linking between illicit drug preparations, consumers and traffickers [5,6,14]. Besides, both impurity and chiral profile may provide a link between starting materials and the illicit drugs synthesized by a clandestine laboratory [5,15]. Chiral analysis in biological fluids can give information regarding consumption and differentiation between illegal drugs or legal pharmaceuticals containing only a single enantiomer [7,16]. For example, the ingestion of dexedrine (*S*-(+)-amphetamine (AM)) used in the treatment of narcolepsy, attention deficit disorders and hyperactivity in children results only in serum concentrations of *S*-(+)-AM in contrast to the ingestion of the illegal AM that leads to both enantiomers [7,8,16].

Furthermore, the presence of these compounds in wastewater has been shown to be a tool for the monitoring drug consumption at a community level (sewage epidemiology) [17,18,19]. In fact, once excreted, residues of chiral drugs reach the aquatic environment mainly through the sewage system as parent compounds and metabolites [20,21]. Concentration of target pharmaceuticals or illicit drug residues in wastewater influent may be used to backcalculate drug consumption for local communities (sewage forensics) [17,22,23,24]; an approach to provide direct quantitative estimates, in a non-invasive manner and in almost real-time [17,24,25]. Since most of these compounds are chiral, the determination of the enantiomeric fraction (EF) can give further information about the use of legal and illegal substances. Furthermore, once in the sewage system these compounds are subject to biotic processes that causes changes in the enantiomeric composition. This information may be used to evaluate the efficiency of the wastewater treatment plant (WWTP) or illegal discharges of sewage since it may be expected that their EF in untreated sewage would differ from the one observed in treated effluents [9,18,26]. Also, information about environmental occurrence and distribution of chiral pharmaceuticals in the environment is important for evaluation of enantio-(eco)toxicity in particular for aquatic organisms [27,28,29]. In this sense, chiral analysis applied to drugs preparations, biological fluids and environmental samples may give information about: distinction between legal and illicit drugs; linking between samples, illegal laboratories, consumers and trafickers; estimation of consumption patterns at community level (sewage epidemiology); identification of manufacturing locations of illicit drugs; illegal discharge of sewage and information about ecotoxicity (Figure 1). Concerning the importance of chiral drug analyses in various forensic contexts, the present work aims to critical discuss the applicability of chiral drug analyses concerning pharmaceuticals and illicit drugs in forensic chemistry regarding biological and environmental matrices. The references search were based in ScienceDirect and ISI Web of Knowledge databases considering articles up to 2017 that comprise biological matrices such as urine, plasma, serum, blood and hair and environmental samples as surface waters, influents and effluents from WWTPs as aquatic environmental matrices.

## 2. Chromatography in Chiral Analyses

The separation of enantiomers is currently carried out using liquid chromatography (LC), gas chromatography (GC), capillary electrophoresis (CE) and supercritical fluids chromatography [15,20,30,31,32,33,34,35,36,37,38,39,40,41,42,43,44]. Chromatographic methods for resolution of enantiomers include indirect and direct methods. The indirect method is based on the reaction of the enantiomers with chiral derivatization reagents (CDRs) and the formation of diastereoisomers with different physico–chemical properties that can be separated by conventional means such as chromatography. The direct method can be achieved by chiral stationary phases (CSPs), mostly applied in LC, or using chiral mobile phase additives. Both GC and LC methods are available in indirect and direct [35,45,46,47,48,49]. However, for GC only few chiral columns are available. Thus, most works with GC used indirect approaches with CDRs, which are then separated by achiral columns. Examples of most used CDRs are *S*-(−)-*N*-(trifluoroacetyl)propyl chloride (S-TPC), *R*-(−)-α-methoxy-α-(trifluoromethyl)phenylacetyl (*R*-MTPCl) and *S*-(−)-*N*-(heptafluorobutyryl)propyl chloride (*S*-HFBPrCl) [46,50]. Regarding direct methods, many types of CSP are available, however the cyclodextrin (CD), Pirkle-type, polysaccharide derivatives, antibiotics-based and polymeric-based are most used [20,51,52,53].

GC and LC methods have been widely used for the enantioselective analysis of various classes of illicit drugs in biological fluids though LC methods are most used concerning environmental samples [20,54]. This is probably due to the high number of commercial columns available for LC and the limit of quantification that LC with mass spectrometer (MS) can be achieved. For GC methods most work uses MS while LC use different detectors as MS, ultraviolet-visible (UV/Vis), diode array (DAD) and fluorescence detectors (FD) (Table 1 and Table 2). 

LC/MS and LC/MS/MS are the most applied techniques to quantify chiral compounds in the environment. Nevertheless, MS detection presents some limitations in the type of elution mode and the additives that can be used. Capillary electrophoresis (CE) has also been used for the separation of enantiomers of toxicological, doping and forensic interest due to its simplicity and inexpensive methodology [55]. In this work methods to quantify a variety of chiral illicit drugs and pharmaceuticals (listed in Table 1) in biological and environmental matrices are reunited. 

## 3. Chiral Analyses in Biological Samples

**Figure 2:**
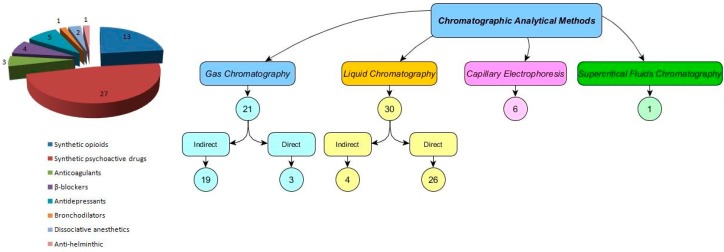
Relative number of each class of chiral drug referred in the reviewed enantioselective published studies and the analytical methods used for separation of the chiral drugs in biological fluids.

This study reviewed 58 articles that have been published between 1996 and 2017 based on ScienceDirect and ISI Web of Knowledge databases. The investigated compounds included synthetic psychoactive drugs (stimulants), synthetic opioids, β-blockers, antidepressants, anticoagulants, bronchodilators and dissociative anesthetics (Table 1 and Table 2). Figure 2 shows the relative number of studies of each class of chiral drug investigated and the analytical methods used for analysis of these compounds in different biological matrices.

The use of racemates typically results in stereoselective pharmacological activity and pharmacokinetic affecting bioavailability, metabolism and excretion that may contribute to the toxicity, increase risk of death or serious adverse effects [11,56,57]. Though there is a tendency for manufacturing pharmaceuticals as single enantiomers, many pharmaceuticals are still supplied as racemates [57]. Concerning illicit drugs, these compounds are also available as racemates or single enantiomers depending on the manufacturing procedure [5,6,57]. Regarding illicit administrations, consumption of pure enantiomer (eutomers), in some cases, may cause overdose or even might lead to lethal cases [10,12]. Thus, significance of chiral analysis has increased since it is possible to determine whether the drug of concern is derived from a controlled or illicit substance [58,59,60]. In fact, some controlled substances are commercialized in the enantiomeric pure form due to their advantages in therapeutic activities [5,6,57]. On the other hand, illicit production of these drugs leads to either racemic or single enantiomers depending on the manufacturing procedure, i.e, racemic or enantiomer pure precursors. Thus, in forensic chemistry, evaluation of the EF may aid in the discrimination of the consumption of legal and illegal substances, give information about method of synthesis used or profile among different seizures linking among them, consumers and traffickers. Chiral analysis can also be applied in doping control, as an example, the use of preparations containing dextromethorphan by athletes is allowed, whereas the use of levorphanol is expressly banned by the International Olympic Committee [61].

Though various works have been published concerning chiral separation of the different classes of pharmaceuticals and illicit drugs of forensic interest in biological matrices most works do not determine the enantiomeric composition. Data about the enantiomeric composition of parent compounds and metabolites is of high importance for accurate data interpretation and for further analysis of results [62]. Also, metabolites of achiral compounds can also be chiral and should be considered in biological samples.

### 3.1. Synthetic Psychoactive Drugs

This class of chiral drugs was the most studied (Table 1, Table 2 and Figure 2). Among synthetic psychoactive drugs are the amphetamine-like drugs, a group of structurally related compounds with vast potential for abuse, addiction and toxicity [63]. Among most studied compounds are AM, methamphetamine (MA), 3,4-methylenedioxymethylamphetamine (MDMA), 3,4-methylenedioxy-ethylamphetamine (MDEA) and methylphenidate (MPH) (Table 1 and Table 2). Enantiomers of these drugs have been discriminated in plasma, urine, blood and hair. Analytical methods used for the separation of enantiomers of these drugs included LC-MS, GC-FID and GC-MS and CE. Amphetamine-like drugs can be used for the treatment of some disorders such as selegiline (L-deprenyl, SG) used for treatment of Parkinson’s disease, Adderall or Elvanse for treatment of attention-deficit hyperactivity disorder or famprofazone, a nonsteroidal anti-inflammatory agent, used for pain control [8,59]. Consumption of SG produces *S*-MA and its metabolite *S*-AM while famprofazone produces both *R*-MA and *S*-MA and their metabolites in human body [7]. Adderall contains both *R*-AM and *S*-AM. On the other hand, these substances are often misused for recreation purposes and even by healthy individuals to enhance work or school performance (e.g., MPH) or doping in sport practice [64,65]. Thus, illicit preparations have been an alternative route of access these substances by consumers and abusers. Illicit production of AM may use 1-phenyl-2-propanone and other reagents such as formic acid, ammonium formate or formamide are used, which is designated as the Leuckart method, yielding a racemic product [17].

Manufacture of MDMA and related drugs can use safrole, isosafrole, piperonal or 3,4-methylenedioxyphenyl-2-propanone (PMK). Many illicit drug syntheses start with PMK and use either the Leuckart route or various reductive aminations, producing a racemic MDMA [17].

Therefore, studies based on determining the ratios of *R*- and *S*-isomers of the parent compounds and metabolites are important for distinctive medical drug administration or illegal abuse of amphetamine like drugs [8]. Also, chiral information is useful and essential to identify the precursor, the synthetic pathway, and intrinsic characteristics of the seized samples [5,6]. In this context, analysis of the enantiomers of AM and metabolites have revealed to be very useful, since it can provide information about the origins of the drug consumed (legal or illicit) [58,60].

In the majority of chiral amphetamine-like drugs, the *S*-enantiomers exhibit greater potency than the *R*-enantiomers [60,66,67,68,69,70].

**Figure 3:**
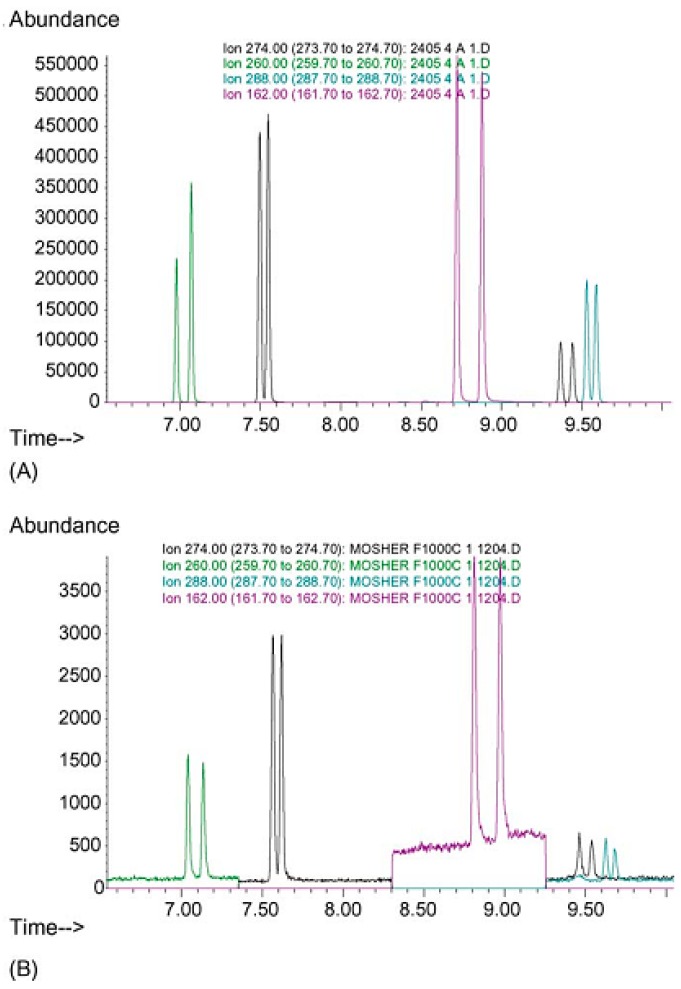
Chromatogram representing the enantioseparation of *R*/*S*-AM, *R*/*S*-MA, *R*/*S*-MDA, *R*/*S*-MDMA and *R*/*S*-MDEA as *R*-MTPCl derivatives in whole blood concentrations at (**A**) 2 µg/g and (**B**) at 0.002 µg/g, respectively. Reproduction with permission of Elsevier (Figure 1 from Rasmussen et al. [67]).

Nishida et al. described a LC-MS method for the determination of the enantiomers of MA, AM, SG and its metabolite, desmethylselegiline (DMSG), in hair samples [59]. In this study, authors showed differences in the enantiomer ratio of MA and AM and between MA abuse consumers and SG consumers [59]. Besides, it was also shown that the existence of DMSG in SG users that is not normally found in urine demonstrating that the method can be useful for distinguish therapeutic users of SG and MA abusers. Fujii et al. described a GC-MS method based on the formation of diastereoisomers using CDR TPC for the separation of the enantiomers of MA, AM, MDMA and MDA in urine samples [8]. This method can be used for discrimination between legal and illegal consumption of these drugs [8]. Rasmussen et al. developed for the first time a GC-MS enantioselective method for the separation of AM, MA, MDA, MDMA and MDEA in whole blood based in the formation of diastereomers [67] (Figure 3).

The method was validated and applied to four whole blood samples from forensic cases including a suspected case of driving under the influence of drugs. In all cases amphetamines were ingested as racemates with stereoselective metabolism since the *R*/*S* ratio for most enantiomers were >1 showing that the *R*-enantiomer is metabolised faster than the *S*-enantiomer. Hädener et al. described a two dimensional LC-MS/MS method for quantification of AM enantiomers in human urine [16]. The study was applied to 67 urine samples from suspected AM abusers, subjects treated with *S*-AM prodrug and suspected MA abusers. In each 40 samples obtained from suspected AM abusers both enantiomers were present and mean *R*/*S* ration was 1.25 indicating a predominance of the *R*-enantiomer. The excepted value *R*/*S* would be 1 but due to stereoselective metabolism of AM in which *S*-AM is metabolized faster than *R*-AM resulting in higher concentrations of *R*-AM. In the consumers of the prodrug it was found only *S*-AM and *R*-AM in one sample at <LOQ probably due to imputities of the drug manufacture itself [16]. Considering individuals suspected of MA consumer, in 80% of the samples, both enantiomers were found although with a predominance of *S*-AM. *R*/*S* ration ranged from 0.01 to 0.47. Five samples from MA abusers contained only *S*-AM. This result was explained by the faster metabolization of *S*-enantiomer of MA. Thus, more *S*-MA is converted to *S*-AM than *R*-MA to *R*-AM.

Binz et al. developed an analytical method for chiral analysis of AM in hair [7]. In this study, analysis of hair samples from nine Elvanse patients revealed only *S*-AM in eight cases. One subject showed both enantiomers indicating a (side-) consumption of street AM. The analysis of the 16 AM abusers samples showed only racemic AM. Furthermore, it could be shown in a controlled study that *S*-AM can be detected after administration of even very low doses of lisdexamfetamine and dexamphetamine, which can be of interest in forensic toxicology and especially in drug-facilitated crime [7].

MPH, another CNS stimulant, is used in the treatment of attention deficit hyperactivity disorder (ADHD) and narcolepsy. Abuse of this substance has been reported [71]. This drug is commercialized in the racemic mixture though only the D*-threo-*form is responsible for the desired therapheutic effect. MPH is enantioselectively metabolized, preferring *S*-MPH over *R*-MPH to ritanilic acid (RA) that is pharmacologically inactive. Individual variations on MPH metabolization classified some individuals as poor metabolizers. Thomsen et al. developed an LC-MS/MS enantioselective method for determination of MPH and RA in femoral blood applied to forensic cases [65] in order to evaluate poor metabolizers by estimating *R*/*S* ratio of MPH. Postmortem blood samples from autopsy cases and antemortem blood samples from mixture of traffic, violence and sexual assault cases were analyzed. Apart from one case, *R*-MPH showed the highest concentration in the postmortem cases, a similar pattern to the found in living organisms. Concentration of RA was higher in all cases than MPH with equal distribution of *R* and *S* enantiomers. In antemortem individuals the same pattern was observed with higher levels of *R*-MPH and equal quantities of *R* and *S* forms of RA.

These reports demonstrate that knowledge about the enantioselective behavior and measure of the enantiomeric ratio of these types of drugs can provide useful and valuable data in the forensic field giving information about consumption of licit and illicit amphetamine like drugs and other psychoactive drugs and essential to aid in the correct interpretation of the use of these substances.

### 3.2. Synthetic Opioids

The second most studied classe of chiral compounds are the synthetic opioids (Table 1 and Table 2). Among reported compounds are tramadol, methadone and methorphan [72,73]. Opioid abuse, addiction, and overdose are considered of a serious public health [54]. In the European Monitoring Centre of Drug and Drug Addiction report of 2017, opioids were the third most consumed class of drugs of abuse in Europe and the first with more fatal cases [74]. Concerning tramadol, it is a synthetic opioid that acts as agonist by selective activity at the µ-opioid receptors commercialized as a racemate of the more active 1*R*,2*R*-enantiomer ((+)-tramadol) and the less active 1*S*,2*S*-tramadol ((−)-tramadol), with both enantiomers acting through different mechanisms, but in a synergistic manner [75]. However, there are differences in their binding properties leading to considerable differences in pharmacological activities [75]. Tramadol is metabolized to *O*-desmethyltramadol (ODT) and *N*-desmethyltramadol (NDT). *O*-Demethylation of tramadol is carried out by CYP2D6; enzyme expressed polymorphically [76]. Polymorphisms play an important role in inter-individual drug response [77]. The metabolite ODT is pharmacologically active, has longer half-life and is more potent than parent compounds. Tramadol has been used for nonmedical purposes due to it euphoric and mood enhancing effects [78,79]. Tramadol abusers develop physiological dependence which can cause negative effects such as convulsion and seizures [80]. Besides, genetic polymorphisms can influence biological properties including toxicity. A LC-MS/MS method for separation of tramadol, and its principal metabolites, ODT and NDT for pharmacokinetic applications in plasma samples was reported [81]. Authors showed that plasma binding was not enantioselective, nevertheless kinetic disposition of tramadol and its NDT metabolite was enantioselective, with plasma accumulation of (+)-tramadol and (+)-NDT, whereas the pharmacokinetics of ODT was not enantioselective in patients with neuropathic pain phenotyped as extensive metabolizers of CYP2D6. Thus, enantioselective methods for both tramadol and its metabolites are essential for an accurate evaluation of their biological properties and toxicity.

Methadone is a synthetic opioid frequently used for treatment of opiate dependent persons, pharmacologically similar to morphine, but lacks the euphoric effects [82,83]. Methadone can be fatal by itself or by interaction with other drugs such as depressors of the CNS. Methadone is a substrate for CYP2B6 and CYP2C19, which are all stereoselective [83]. Plus, CYP P450 isoenzymes are known to have individual variability (polymorphisms) that leads to poor metabolizers, rapid metabolizers and ultra-rapid metabolizers [83]. This chiral drug, when administred as racemate gives rise to enantiomeric metabolites *S*-(−)-2-ethylidine-1,5-dimethyl-3,3-diphenylpyrrolidine (EDDP) and the *R*-(+)-enantiomer. The *R*-(−)-enantiomer of methadone, is the one with higher affinity for the μ-opioid receptor having a higher analgesic potency (over fifty times more) [82,83]. On the other hand, the *S*-enantiomer is responsible for the poor cardiac tolerance [84]. In order to investigate the enantiomeric ratios of methadone and EDDP in postmortem samples, Jantos et al. applied a LC-MS/MS method in femoral blood, urine, bile, brain, lungs, kidneys and muscle tissue samples [82]. The study was based in sixteen samples, from eleven man and five women with ages ranging between 23 to 43 years old. Concentrations of *R*-methadone and *R*-EDDP were found in all body fluids and tissues, while *S*-enantiomers were only found in thirteen of the cases. The *R*/*S* ratios ranged from 0.58 to 4.19 for methadone, and from 0.38 to 1.38 for EDDP [82]. Because methadone does not suffer racemization in the human body, the enantiomeric ratios found in postmortem samples, can reflect if the substance consumed was either racemic or not; also can identify if the cause of death is related to toxic exposure [82]. Moody et al. developed an enantioselective method by LC-MS/MS for methadone and EDDP in human plasma, urine and liver microsomes [85]. This study demonstrated differences in the pharmacokinetic between enantiomers of methadone and its main metabolite EDDP and suggest greater production of and lesser clearance of *S*-EDDP.

The antitussive dextromethorphan (allowed drug) and the narcotic analgesic levomethorphan (banned drug, not commercially available) are the *R*- and *R*- isomers of 3-methoxy-*N*-methylmorphinan. Aumatell and Wells developed a CE chiral method for separation of methorphan (racemate of dextromethorphan and levomethorphan) [86]. Distinction of these compounds is not only of interest in forensic science (such as the elucidation of the cause of death after intake of levomethorphan), but also for the treatment of intoxicated patients. Also, the use of preparations containing dextromethorphan by athletes is allowed, whereas the use of levorphanol is expressly banned by the International Olympic Committee [61].

### 3.3. Antidepressants

Although antidepressants are considered non-addictive, many people abuse of these drugs [87,88]. Users can become physically dependent and non-compliance may arise as a result, with fatal consequences in some cases [89]. Among studied antidepressants are fluoxetine (FLX), citalopram, reboxetine, venlafaxine (VNF) and its metabolites (Table 1 and Table 2). FLX is an example of antidepressants administrated as racemate, that both enantiomers have the same biological active. It acts by selective inhibition of the serotonin reuptake pump, increasing the extracellular catecholamines, such as serotonine, dopamine and norepinephrine. In the human body, it is metabolized to norfluoxetine (NFLX). FLX enantiomers are approximately equipotent in blocking the 5-HT reuptake, while the enantiomers of NFLX show marked differences in pharmacological activity. The enantiomer *S*-NFLX shows approximately 20 times more potency than the *R*-enantiomer as 5-HT reuptake inhibitor, both in vitro and in vivo [90,91,92]. Shen et al. considered the enantioseparation of FLX in human plasma but its metabolite was not considered [93]. Nevertheless, Unceta et al. developed an LC-FD method for simultaneous separation of FLX and NFLX enantiomers, in order to investigate potential sources of variability, in rats receiving chronic treatments, on concentrations of FLX and NFLX and their enantiomers [91]. The plasma levels of *R*-NFLX were considerably increased in comparison to the *S*-enantiomer. In plasma FLX *R*/*S* ratios were of 1.02 compared to 1.05 in cerebral cortex, which was in contrast with NFLX *R*/*S* ratios, that were 1.81 in plasma and 1.5 in cerebral cortex [91].

Citalopram, used in the treatment of depression, commercialized as racemate and its enantiomer *S*-(+)-citalopram (escitalopram, marketed in the enantiomerically pure form) is 100 times more potent as a serotonin reuptake inhibitor as compared to *R*-(−)-citalopram.

VNF is a phenylethylamine derivative that affects brain neurotransmission by blocking presynaptic reuptake of serotonin and noradrenaline [94], and administrated in the treatment of psychiatric disorders [95]. VNF undergoes extensive first-pass metabolism by CYP P450 enzymes into its major active metabolite *O*-desmethylvenlafaxine (*O*D-VNF), and two minor metabolites, *N*-desmethylvenlafaxine (*N*D-VNF) and *N*,*O*-didesmethylvenlafaxine (*N,O*-DD-VNF). *O*D-VNF inhibits the reuptake of serotonin and noradrenaline in similar potency to that of VNF [96]. Stereoselective metabolism has been observed both in vitro and in vivo, where CYPD2D6 displays and appreciable stereoselectivity towards the *R*-enantiomer [96].

Reboxetine is used as a selective noradrenaline reuptake inhibitor for the treatment of major depressive disorders, commercialized as racemate (*S*,*S*- and *R*,*R*-reboxetine). Ohman et al. developed an enantioseletive method for analysis of reboxetine in serum in patients with chronic medication [97]. Authors found that the median *S*,*S*/*R*,*R* ratio in steady state was 0.5 and ranged from 0.22 to 0.88. It was also shown that women have an approximately 30% higher *S*,*S*/*R*,*R* ratio than men. The *S*,*S*/*R*,*R* ratios of reboxetine were not found to correlate with reboxetine concentrations. Authors also found a correlation between selective noradrenaline reuptake inhibitor activity that is higher in women than in men and that may alter the enantiomeric ratio.

### 3.4. β-Blockers

β-Blockers, also known as β-adrenergic blocking agents, are a class of chiral drugs that are used for the management of cardiac arrhythmias. Usually one enantiomer presents higher potency than the other. For instances, *S*-(−)-propranolol (PHO) is 100 times more than *R*-(+)-PHO. Most of β-blockers (except timolol: *S*-isomer) are marketed as racemates, such as acebutolol, atenolol (ATE), alprenolol, betaxolol, carvediol, metoprolol (MET), labetalol, pindolol and sotalol [4]. In addition to therapeutic properties, these compounds exhibit calming neurological effects decreasing anxiety, nervousness and stabilizing motor performance. Thus, these compounds are included in prohibited list according to the World Anti Doping Agency (WADA) regulation because of the improved psychomotor performance that may be beneficial in sports requiring precision and accuracy such as shooting archery among others [61]. Among β-blockers only PHO, MET, carvediol, verapamil and its metabolite enantiomers were discriminated in plasma and urine (Table 1 and Table 2) [98,99,100,101]. Analytical methods used for the separation of enantiomers of these drugs included LC-MS and GC-MS. PHO is administered as racemate to treat hypertension and normalize tachycardia response; however, the *S*-enantiomer shows greater cardiosympatholytic activity [102]. Siluk et al. developed an analytical method for separation of *R*,*S*-PHO in human plasma for determination of pharmacokinetic difference among the two enantiomers and even drugs interaction [98]. In this study, authors suggest that *R*-PHO is eliminated faster than *S*-PHO. Concerning MET, Kim et al. developed an analytical method for enantioseparation of its enantiomers in urine. This method can be applied in pharmacological and pharmacokinetic studies of both enantiomers in biological samples [100]. Beyond carvediol and verapamil, there are not studies concerning the enantioseparation of other used β-blockers in biological samples. Methods of enantioseparation for these substances are important to evaluate pharmacological and pharmacokinetic differences among enantiomers and possible toxicity due to interaction with other administered drugs.

### 3.5. Anticoagulants

Warfarin (WFN) is one of the most commonly prescribed cardiovascular medication anticoagulant drugs used to manage thromboembolic disease. WFN is administered as an oral medication consisting of a racemate though the *S*-enantiomer has higher activity than the *R*-enantiomer. Several factors increase the risk of over-anticoagulation such as genetic polymorphisms as well as others factors, including age, sex, and histories of smoking and alcohol consumption and diets rich in vitamin K [103]. Genetic factors and drug interactions mostly account for the risk of over-anticoagulation [103]. Knowledge about enantioselective pharmacodynamic and pharmacokinetic is not only important to assure efficiency and safety but also because genetic polymorphisms may have an important impact in biological properties including toxicity. Separation of WFN enantiomers was achieved using different analytical methods: SFC-MS/MS, LC-MS/MS and Micellar electrokinetic chromatography MEKC-MS in plasma (Table 1 and Table 2) [104,105,106]. Knowledge about pharmacokinetic of enantiomers of WFN and its metabolites may add in the development of enantiopure commercialized forms of WFN that may be safer and for studies of possible toxicological and interaction of WFN with other pharmaceuticals concomitant administered. Beyond the use of WFN as anticoagulant, this compound was used as a poison, and is still marketed as a pesticide against rats and mices.

### 3.6. Dissociative Anaesthetics

Ketamine (K) began to be a widespread drug of abuse in many countries and primarily available through illicit means. At sub anaesthetic doses this drug provides hallucinogenic effects [107,108]. Because of these desire effects K is often used in recreational purposes and particularly dangerous with regards to traffic and workplace safety. In fact, K can be bought in the internet from suspected veterinary distributers and clinics [109]. Chiral discrimination of K and its main metabolite norketamine (NK) was done in in plasma and hair [110,111]. *S*-(+)-K is an anesthetic and analgesic but *R*-(−)-ketamine is associated to hallucinations and agitation. K is a dissociative anaesthetic that induces loss of consciousness, amnesia, immobility, and in a lesser extent analgesia [110]. It is used in paediatric emergency retrieval and in veterinary surgery, because of its reduced tendency to give respiratory depression [110]. Its main advantage is to induce profound analgesia and amnesia, while maintaining the cardiopulmonary functions and the protective airway reflexes stable [110]. K undergoes extensive first-pass metabolism to produce various free and glucurinated hydroxylated derivatives [110]. However, its main metabolic pathway occurs through *N*-demethylation to NK which appears to have 20–30% activity of its parent drug [110]. Although is used as a racemate, the *S*-enantiomer showed to have four times higher affinity for the phencyclidine site of the NMDA receptor, as well as a greater potency when compare to the *R*-form and the racemate [110].

### 3.7. Bronchodilators

Among bronchodilators are β-adrenoreceptor agonists (or β2-agonists) that are drugs commonly used for the treatment of asthma and other pulmonary disorders. They have bronchodilator and anabolic activities. Because of these properties, these compounds may be used by athletes to enhance performance and as a safer alternative to anabolic steroids though the use by asthmatic athletes is not forbidden. In this sense, like β-blockers, the β-adrenergic compounds are scheduled in the Prohibit List of the WADA [61]. Only one report was found that describes the enantioseparation of a bronchodilator, the salbutamol (SBT). This compound is commercialized as racemate, however the *R*-enantiomer of SBT binds to β2-adrenergic receptors with greater affinity than the *S*-enantiomer, which does not act through β-adrenergic receptor activation. *S*-SBT has adverse effects associated, such like augmentation of bronchospasm and pro-inflammatory activities. Studies have reported that the *S*-enantiomer can potentiate the effects of spasmogens in airway of smooth muscle from both guinea pigs and humans, with a number of clinical studies also reporting worsening of airways hyper-responsiveness in animals and in subjects with asthma [112]. The initial step in metabolism of both enantiomers is sulfate conjugation, a stereoselective process that occurs in human airway epithelial cells, as also in other cells and tissues [113]. The greater rate of sulfate conjugation of *R*-SBT might lead to lower plasma levels of *R*- than *S*-enantiomer in human subjects, which can be responsible for increasing the adverse effects related with the latter [112,113]. Since bronchodilator pharmacodynamic is enantioselective the development of enantioselective methods for bronchodilators is essential for stereo-pharmacokinetics and enantioselective safety studies. Data from pharmacokinetic studies can contribute to the development of enantiopure broncodilators therapeutic drugs that can be safer and used in the control of broncodilators abuse.

### 3.8. Anti-Helmintic

Levamisole and dexamisol [(phenyltetrahydroimidazothiazole (PTHIT)] were widely used as an anti-worm medication for both humans and animals, but they are no longer approved for use in the United States or Canada due to their toxicity. In South America, illicit cocaine laboratories have been known to add PTHIT to cocaine preparations to extend their effects [10]. Casale et al. developed an analytical method by GC-FID for the determination of PTHIT enantiomers. i.e., levamisole and dexamisole in illicit cocaine seizures and in urine cocaine abusers [10]. Beyond the possibility of linking origin of cocaine seizures, the addiction of PTHIT have been shown to cause agranulocytosis/neutropenia in cocaine abusers expanding the adverse effects of the consumption of this drug. Approximately 78% of cocaine samples contained PTHIT in an average concentration of 23%. Enantiomeric compositions of dexamisole/levamisole were different among samples. 66% of the samples contained levamisole, 19% the racemate and 15% the higher levels of levamisole. Samples containing only dexamisole were not detected. The higher content in levamisole may be due to traffickers adding tetramisole and levamisole to the cocaine or illegitimate preparation of levamisole. Considering urine samples the majority of urine extracts contained levamisole (46%), and levamisole enhanced enrichment (20%) and dexamisole-enhanced enrichment (26%). Levamisole has high toxicity affecting all organ systems, with agranulocytosis, manifested primarily as acute, profound neutropenia and have been found in concaine consumers leading to serious clinical complications. Thus, this methodology allowed clinical toxicologists and forensic chemists to establish specific enantiomer composition of PTHIT in cocaine samples and in the urine specimens of cocaine abusers [10]

## 4. Chiral Analyses in the Aquatic Environment

**Figure 4:**
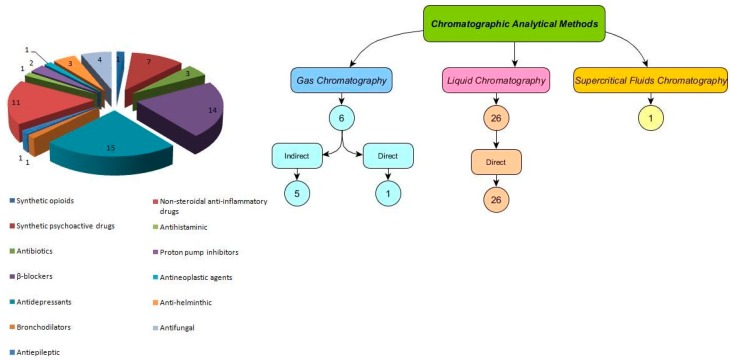
Relative number of each class of chiral drug referred in the reviewed enantioselective published studies and the analytical methods for separation of the chiral drugs in environmental samples.

This study reviewed 33 articles that have been published between 2005 and 2017 based in ScienceDirect and ISI web of Knowledge databases (Table 3 and Figure 4). The target compounds included antidepressants, β-blockers, nonsteroidal anti-inflamatory drugs (NSAIDs), synthetic psychoactive drugs, antibiotics, synthetic opioids, antiepileptics, antihistaminic; bronchodilators, antineoplastic agents and proton pump inhibitors (Table 1). Figure 4 shows the relative number of studies of each class of chiral drug investigated and the analytical methods used for analysis of these compounds in different environmental matrices.

The entrance of pharmaceuticals and illicit drugs into the aquatic environment may occur through effluents from WWTPs which are unable to totally remove these micro-pollutants or direct discharged of sewage. WWTPs biological treatments, can alter the EF of the enantiomers present in the influent, as microbiota action generally is stereoselective [136,137]. Disposed drugs will usually be found in their parent form, either as racemate or single enantiomers. On the other hand, excreted drugs will be normally found as metabolites, frequently chiral, of the parent compound [138].

The possible adverse effects of the enantiomers on aquatic and human life lead to the studies of occurrence of chiral drugs in environmental matrices [20,136,137,139,140]. Illicit drugs can also be found in environmental samples, and environmental data are important resources for a forensic approach. This includes the usage of environmental data in order to: (1) verify patterns of illicit and prescribed drugs usage in local communities (2) application of drugs as chemical markers of faecal water contamination with (human) sewage and (3) verify the source of drugs (legal or illicit) [20,136,137,139,140].

The estimation of the consumption of substances of abuse and illicit drugs can be measured by the concentrations of these compounds in wastewater. Drugs are consumed and metabolised in human body and excreted as parent compounds or as metabolites, and finally reach WWTPs through the sewage [139]. Since the metabolic patterns of most available drugs are understood, it is assumed that the amount of drug or its metabolite quantified in raw sewage will correspond with the consumed dose—sewage epidemiology [139]. The application of the chiral discrimination has also been used for distinction between legal and illicit use of drugs, verification of the method of synthesis of illicit drugs, identification that drug residue results either from consumption of illicit drugs or metabolism of other drugs, verification of route of administration, verification of potency of abused drugs, monitoring of changing patterns of drugs abused, and differentiation between consumption and disposal of unused drugs [17,141].

Concerning biodegradation, ecotoxicity and environmental fate, the recognition of enantioselectivity is essential to provide a more realistic risk assessment of chiral compounds. The fate of chiral drugs in the environment can be studied by monitoring their EF during biological processes [20,38,136]. Degradation of these compounds relies on both abiotic and biotic processes [142]. Biodegradation in WWTP is expected to be stereoselective, which changes the EF of a given molecule in the sample, consequently bringing different removals/degradation rates [142]. Over the past five years, the amount of research published in chiral environmental analysis has been increasing on a high rate. Knowledge on how chiral micropollutant, such as pharmaceuticals and illicit drugs, behave in the environment, especially in water samples, either wastewater or superficial water, has been providing valuable information, both for risk assessment and WWTPs efficiency [139,142]. The EF of certain pharmaceuticals, such as PHO, alprenolol, VNF and climbazole [136,143,144] in surface waters, can reveal the efficiency of different WWTPs [136,139,140,143,144,145,146]. Additional these compounds have been pointed as indicators to differentiate between treated and untreated water. Analysis of wastewater samples are mostly done by comparing the EF of the influent and effluent of the target analytes, which gives an overview of the efficiency and of the WWTPs [9,147,148].

According to Kasprzyk-Hordern et al., since WWTPs are fed by fresh sewage, a long-term monitoring programme of drugs might reveal their usage patterns in local communities and their changes over longer periods of time [139]. This is the main route that chiral drugs enter the environment, and these can be found either in a modified form (metabolites) and/or with alterations in their enantiomeric EF due to human metabolism [138]. In the first attempt to apply chirality to sewage epidemiology, Kasprzyk-Hordern et al., collected wastewater samples over a period of 8 months, from seven WWTPs in London, during five sampling campaigns and, quantified the levels of AM, MA, MDMA, MDA, ephedrine and pseudoephedrine enantiomers [149]. The samples were enriched with *R*-AM, *S*-MA, *S*-MDA, 1*R*,2*S*-(−)-ephedrine and 1*S*,2*S*-(+)-pseudoephedrine. However, the authors could not reach any conclusion according to the use of illicit drugs, since AM and MA enantiomers can also result from the metabolism of chiral pharmaceuticals. On the other hand, when comes to MDMA and MDA, the enantiomeric profiling proved to be invaluable in making distinction between MDA abuse and its formation due to metabolism of MDMA, suggesting that this profiling could also help with making a distinction between actual consumption and direct disposal [17].

Vasquez-Roig et al., in a two week study of three WWTPs located in the city of Valencia (Spain) and surroundings, described the enantiomeric profile of some chiral drugs [148]. Although for some of the target analytes it was not possible to study their enantiomeric fate, since these were present in very low concentrations, which was the case of MDMA and AM, they were able to observe enantiomeric enrichment ofATE, where the *S*-enantiomer was in higher abundance in raw wastewater, meanwhile during the wastewater treatment, enrichment of both *R*- or *S*-enantiomer were observed [148]. This difference in enantiomeric enrichment seemed to be related with the technology used by the treatment plant. Although all of three used activated sludge, one of the plants had also biological nitrogen removal, which the authors believe that different bacteria were involved in this process (in aerobic conditions), that could favour the degradation of *R*-ATE, leading to an enrichment of *S*-ATE [148]. They also found enrichment at similar levels of 1*R*,2*S*-(−)-ephedrine and 1*S*,2*S*-(+)-pseudoephedrine in raw wastewater. In terms of elimination rates these ranged from 29 to 100% and showed to be compound and enantiomer dependent. AM and MA were not detected in effluents, however stereoselective degradation was observed for MDMA, where the *S*-enantiomer was more readily degraded than the *R*-MDMA. Atenolol was found to be poorly removed, thus *S*-atenolol removal efficiency was higher than *R*-atenolol [148]. VNF concentrations increased in two of the WWTPs after sewage treatment, which in according to the authors, was due to biotic effects, such like, elimination of glucuronide metabolites, back-reversion of the demethylated metabolite, or desorption from particulate matter [148]. The application of wastewater enantiomeric profiling revealed usage patterns of chiral drugs in the region, where the consumption of AM showed an irregular pattern throughout the two-week sampling campaign, while MA showed a slight increase in daily loads, throughout the weekend in one of the WWTPs. MDMA showed a clear weekly pattern of increased daily loads, during weekends [148].

**Figure 5:**
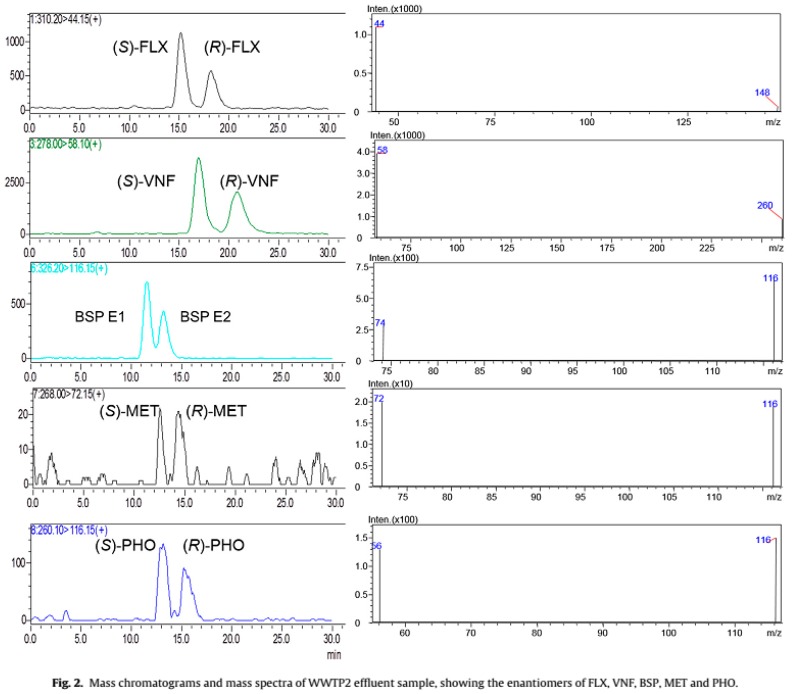
Chromatogram and mass spectra of WWTP effluent sample showing the enantiomers of FLX, VNF, BSP, MET and PHO. Reproduction with permission of Elsevier (Figure 2 from Ribeiro et al. [39]).

Hashim, N. H. & Khan, S. J. studied the EFof ibuprofen, naproxen and ketoprofen in wastewater samples, taken from a WWTPs in Sydney (Australia) with tertiary treatment over seven separate sampling events, during June and August 2010 [147]. For ibuprofen, EF ranged from 0.49 and 0.62; 0.66 and 0.86 for naproxen and 0.54 and 0.66 for ketoprofen [147]. Also Barreiro et al in 2010, found for the first time the occurrence of (+)-omeprazole and (−)-omeprazole, while simultaneously developing a column switching, liquid chromatography method for the chiral separation of these drugs, in wastewater and estuarine samples [150]. Ribeiro et al studied, the EF of FLX, NFLX, VNF, SBT, alprenolol, MET, PHO and bisoprolol (BSP = from the final effluent of the secondary clarifier of three WWTPs located in the North of Portugal, Figure 5 [39]. Regarding antidepressants, only *R*-FLX was detected in two of the WWTPs, indicating a faster degradation of the *S*-enantiomer during the biological degradation [39]. VNF enantiomers were found between 40.4 and 129 ng/L in the three WWTPs studied, with similar EF, which varied between 0.54 and 0.55, proving that VNF found was not racemic [39]. Concerning the β-blockers enantiomers, BSP and PHO were found in all three WWTPs, while MET was only found in two, however all of them under the medium quantification level (MQL) [39]. 

## 5. General Conclusions and Further Perspectives

The LC-MS/MS is the first choice for environmental and biological matrices analyses due to the low quantification limits, the selectivity and unequivocal identification. Regarding environmental analysis the direct method by LC using CSP are mostly described. However methods for a complex mixture of chiral drugs are still scarce. Chiral analyses in biological matrices describe many indirect methods by GC, but the trend is the direct method by LC.

Despite the importance of the chiral analysis in forensic chemistry, this type of data are not yet currently in used in certificated laboratories for doping control, criminal offense, environmental monitoring and chiral drug control in general. In this sense more research is needed regarding new enantioselectivity methods with different CSP and demonstrations with practical applications to establish the importance of the chiral analysis in forensic chemistry.

## Figures and Tables

**Table 1:** Structures of the chiral illicit drugs and pharmaceuticals.

Chiral Compounds		
**Stimulants and their metabolites**		
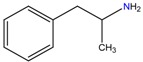	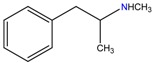	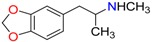
Amphetamine (AM)	Metamphetamine (MA)	3,4-Methylenedioxy-methamphetamine (MDMA)
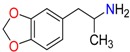	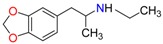	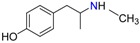
3,4-Methylenedioxy amphetamine (MDA)	Methylenedioxy-*N*-ethylamphetamine (MDEA)	*p*-Hydroxymethamphetamine (pOHMA)
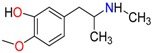	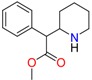	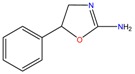
4-Hydroxy-3-methoxy methamphetamine (HMMA)	Methylphenidate (MPH)	Aminorex
**Drug precursors**		
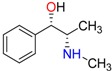	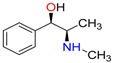	
Pseudoephedrine	Ephedrine	
**Opioids, morphine derivatives and their metabolites**		
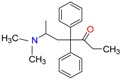	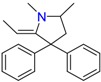	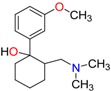
Methadone	2-Ethylidine-1,5-dimethyl-3,3-diphenylpyrrolidine (EDDP)	Tramadol (T)
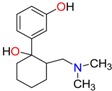	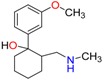	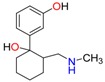
*O*-Desmethyltramadol (ODT)	*N*-Desmethyltramadol (NDT)	*N*,*O*-Ddidesmethyltramadol (*N,O*-DDM-T)
**Antidepressants and their metabolites/Benzodiazepine**		
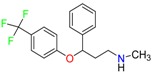	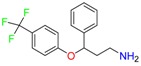	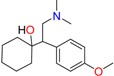
Fluoxetine (FLX)	Norfluoxetine (NFLX)	Venlafaxine (VNF)
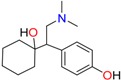	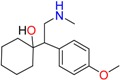	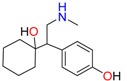
*O*-Desmethylvenlafaxine (*O*-DES-VNF)	*N*-Desmethylvenlafaxine (*N*-DES-VNF)	*N*,*O*-Didesmethylvenlafaxine (*N,O*-DES-VNF)
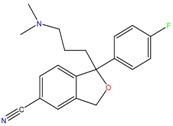	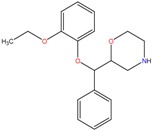	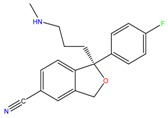
Citalopram	Reboxetine	D-citalopram
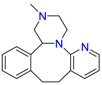	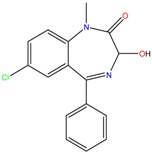	
Mirtazapine	Temazepam	
**Dissociative anaesthetic and its metabolite**		
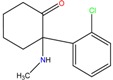	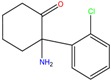	
Ketamine	Norketamine	
***β*-Blockers and anti-arrhythmic**		
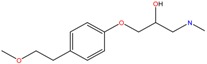	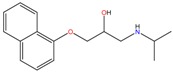	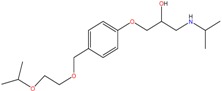
Metoprolol (MET)	Propranolol (PHO)	Bisoprolol (BSP)
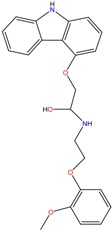	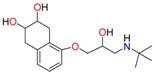	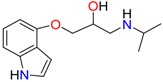
Carvedilol	Nadolol	Pindolol
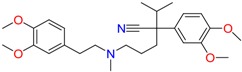	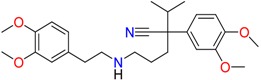	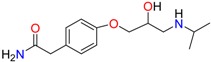
Verapamil	Norverapamil	Atenolol
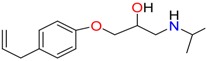	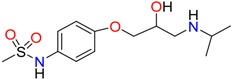	
Alprenolol	Sotalol	
**Anticoagulants**		
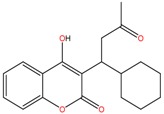		
Warfarin (WFN)		
**Non-steroidal anti-inflamatory drugs**		
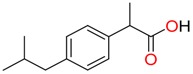	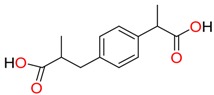	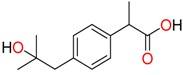
Ibuprofen	Carboxyibuprofen	2-Hydroxyibuprofen
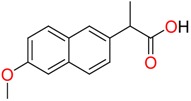	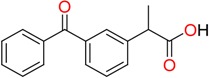	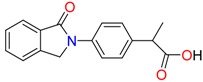
Naproxen	Ketoprofen	Indoprofen
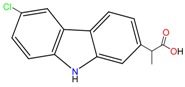	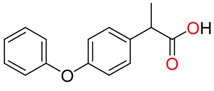	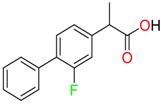
Carprofen	Fenoprofen	Flurbiprofen
**Bronchodilators**		
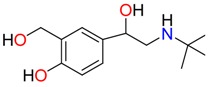		
Salbutamol (SBT)		
**Antibiotics**		
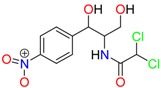	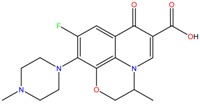	
Chloranfenicol	Ofloxacin	
**Antiepileptic and their metabolites**		
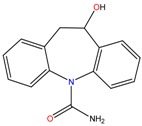		
10,11-Dihydro-10-hydroxycarbamazepine		
**Proton pump inhibitors**		
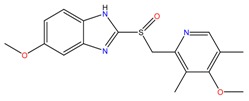	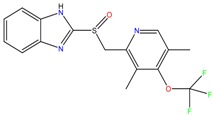	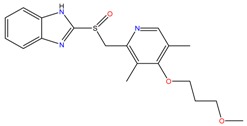
Omeprazol	Lanzoprazol	Rabeprazole
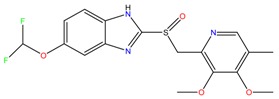		
Pantoprazole		
**Antineoplastic agents and their metabolite**		
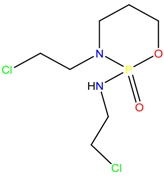	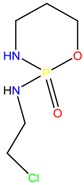	
Ifosfamide	3-*N*-Dechloroethylifosfamide	
**Antifungals**		
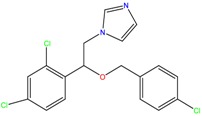	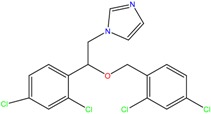	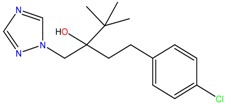
Econazole	Miconazole	Tebuconazole
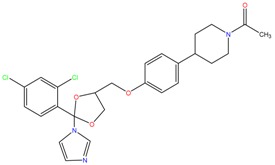	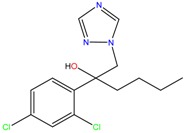	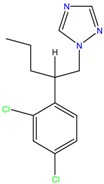
Ketoconazole	Hexaconazole	Penconazole
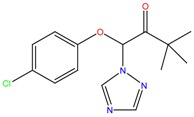	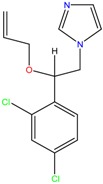	
Triadimefon	Imazalil	
**Anti-helminthic**		
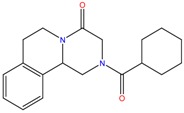	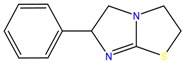	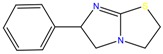
Praziquantel	Tetramisole	Phenyltetrahydroimidazo thiazole (PTHIT, levamisole/dexamisole)
**Antihistaminic**		
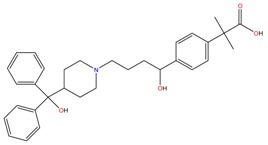		
Fexofenadine		

**Table 2:** Chromatographic analytical methods described for the analysis of chiral illicit drugs and pharmaceuticals in biological matrices.

Drug	Matrix Application	Method	Stationary Phase	LOD	LOQ	Concentration Range/ER (When Mencioned)	Reference
**AM**	Plasma	GC/NICI-MS	SGE-BPX5 (15 m × 0.25 mm, 0.25 µm film thickness)	50 fg	0.049 ng/mL (*R*) 0.195 ng/mL (*S*)	0.006–50 ng/mL	[30]
			HP-5MS (30 m × 0.25 mm, 0.25 µm film thickness)	n.r.	5 µg/L	5–250 µg/L; ER (*R*/*S*): 0.97–1.66, with a mean value of 1.15	[114]
		GC/EI-MS	HP-5MS (30 m × 0.25 mm, 0.25 µm film thickness)	n.r.	5 ng/g plasma	5–400 ng/g plasma	[31]
	Blood	GC/EI-MS	HP-5MS (30 m × 0.25 mm, 0.25 µm film thickness)	n.r.	0.004 µg/g	0.004–3 µg/g	[67]
		GC/NICI-MS	HP-5MS (30 m × 0.25 mm, 0.25 µm film thickness)	0.8 pg/mg (*R*) 0.7 pg/mg (*S*)	2.7 pg/mg (*R*) 2.4 pg/mg (*S*)	0.003–60 ng/mg (*R*) 0.002–60 ng/mg (*S*) ER (*R*/*S*): 0.03–0.95	[49]
	Hair	GC/EI-MS	HP5-MS (30 m × 0.25 mm, 0.25 µm film thickness)	n.r.	2.5 ng/sample	2.5–100 ng/sample	[31]
			5% phenyl-methylsilicone capillary column (17 m × 0.2 mm, 0.33 µm film thickness)	0.1 ng/mg	0.2 ng/mg	0.2–20 ng/mg	[69]
		LC/MS/MS	n.r.	20 pg/mg	50 pg/mg	50–20000 pg/mg	[7]
		HPLC/ESI-MS	Chiral DRUG (150 mm × 2 mm)	0.05 ng/mg	n.r.	0.2–40 ng/mg	[59]
	Urine	GC/EI-MS	DB-5MS (20 m × 0.18 mm × 0.18 mm)	10 ng/mL	n.r.	25–10000 ng/mL	[115]
			HP-5MS (20 m × 0.25 mm, 0.25 µm film thickness)	n.r.	10 µg/L	10–500 µg/L	[42]
			HP-5MS (30 m × 0.2 mm, 0.33 µm film thickness)	40 ng/mL	45 ng/mL	45–1000 (*l*; *d*) 45–2000 (*d*,*l*)	[70]
			5% phenyl polysiloxane (15 m × 0.2 mm, 0.2 µm df)	n.r.	n.r.	25–10000 ng/mL	[116]
		GC/MS	HP-5MS (20 m × 0.25 mm, 0.25 µm film thickness)	1.1 ng/mL (*R*) 1.3 ng/mL (*S*)	3.7 ng/mL (*R*) 4.3 ng/mL (*S*)	5–500 µg/L	[117]
			DB-5 (10 m × 0.1 mm, 0.4 µm film thickness)	0.5 ng/mL	n.r.	20–1000 ng/mL	[8]
		CE/ESI-MS	Uncoated fused silica capillary (50 µm, 100 cm)	0.02 µg/mL	n.r.	0.05–10 µg/mL	[33]
				0.03 µg/mL	n.r.	0.2–10 µg/mL (*S*)	[118]
		CE	PVA chemically modified diol capillary column (40 cm × 50 µm)	n.r.	n.r.	n.r.	[36]
		LC/MS-MS	Lux AMP (150 × 3 mm, 5 µm)	n.r.	0.05 mg/L	0.05–25 mg/L	[16]
			Chirobiotic V2 (250 × 2.1 mm, 5 µm)	0.02 mg/L	0.05 mg/L	0.05–50.00 mg/L	[60]
		HPLC-UV	Adsorbosphere HS, C18 (150 × 4.6 mm, 5µm); C18 precolumn (7.5 × 4.6 mm)	n.r.	n.r.	0.1–100 mg/L	[119]
**MA**	Plasma	GC/NICI-MS	HP-5MS (30 m × 0.25 mm, 0.25 µm film thickness)	n.r.	5 µg/L	5–250 µg/L ER (R/S): 1.02–1.63, with a mean value of 1.33	[114]
	Blood	GC/EI-MS	HP-5MS (30 m × 0.25 mm, 0.25 µm film thickness)	n.r.	0.004 µg/g	0.004–3 µg/g	[67]
	Hair	GC/NICI-MS	HP-5MS (30 m × 0.25 mm, 0.25 µm film thickness)	2.1 pg/mg (*R*) 1.5 pg/mg (*S*)	6.9 pg/mg (*R*) 5.0 pg/mg (*S*)	0.007–60 ng/mg (*R*) 0.005–60 ng/mg (*S*) ER (R/S): 0.01–0.82	[49]
		GC/MS	5% phenyl-methylsilicone capillary column (17 m × 0.2 mm i.d., 0.33 µm film thickness)	0.1 ng/mg	0.2 ng/mg	0.2–20 ng/mg	[69]
		HPLC/ESI-MS	Chiral DRUG (150 mm × 2 mm)	0.01 ng/mg	n.r.	0.04–40 ng/mg	[59]
	Urine	GC/EI-MS	DB-5MS (20 m × 0.18 mm i.d. × 0.18 mm)	10 ng/mL	n.r.	25–10000 ng/mL	[115]
			HP-5MS (30 m × 0.2 mm, 0.33 µm film thickness)	40 ng/mL	45 ng/mL	45–1000 (*l*; *d*) 45–2000 (*d*,*l*)	[70]
			HP-5MS (20 m × 0.25 mm, 0.25 µm film thickness)	n.r.	10 µg/L	10–500 µg/L	[42]
			5% phenyl polysiloxane (15 m × 0.25 mm, 0.2 µm df)	n.r.	n.r.	25–10000 ng/mL	[116]
		GC/MS	HP-1 (12 m × 0.25 mm, 0.25 µm film thickness)	n.r.	10 ng/mL	10–2000 ng/ mL	[120]
			HP-5MS (20 m × 0.25 mm, 0.25 µm film thickness)	2.0 ng/mL (*R*) 1.6 ng/mL (*S*)	6.8 ng/mL (*R*) 5.2 ng/mL (*S*)	5–500 µg/L	[117]
			DB-5 (10 m × 0.1 mm, 0.4 µm film thickness)	3 ng/mL	n.r.	20–1000 ng/mL	[8]
		CE/ESI-MS	Uncoated fused silica capillary (50 µm, 100 cm)	0.02 µg/mL	n.r.	0.05–10 µg/mL	[33]
				0.03 µg/mL	n.r.	0.2–10 µg/mL (*S*)	[118]
		CE	PVA chemically modified diol capillary column (40 cm × 50 µm)	n.r.	n.r.	n.r.	[36]
		LC/MS/MS	Chirobiotic V2 (250 × 2.1 mm, 5 µm)	0.02 mg/L	0.05 mg/L	0.05–50.00 mg/L	[60]
		HPLC-UV	Adsorbosphere HS, C18 (150 × 4.6 mm, 5µm); C18 precolumn (7.5 × 4.6 mm)	n.r.	n.r.	0.1–100 mg/L	[119]
**MDMA**	Plasma	HPLC-DAD	ODS-1 (150 mm × 4.6 mm) with precolumn (20 × 4.0 mm)	n.r.	7 ng/mL	n.r.	[121]
	Blood	GC/EI-MS	HP-5MS (30 m × 0.25 mm, 0.25 µm film thickness)	n.r.	0.004 µg/g	0.004–3 µg/g	[67]
		LC/MS/MS	Kinetex C18 column (100 × 2.1 mm, 2.6 μm film thickness)	n.r.	0.0025 µg/L	0.0025–1.25 µg/L (R, S)	[122]
	Hair	GC/NICI-MS	HP-5MS (30 m × 0.25 mm, 0.25 µm film thickness)	1.7 pg/mg (*R*) 1.5 pg/mg (*S*)	5.6 pg/mg (*R*) 5.1 pg/mg (*S*)	0.006–60 ng/mg (*R*) 0.005–60 ng/mg (*S*)	[49]
		GC/MS	5% phenyl-methylsilicone capillary column (17 m × 0.2 mm, 0.33 µm film thickness)	0.2 ng/mg	0.5 ng/mg	0.5–20 ng/mg	[69]
	Urine	GC/NICI-MS LC/HRMS	n.r. (chiral derivatization *S*-HFBPrCl) Chirex3012 (250 × 4.6 mm, 5 µm film thickness)	n.r.	n.r.	n.r.	[68]
		GC/EI-MS	HP-5MS (20 m × 0.25 mm, 0.25 µm film thickness)	n.r.	10 µg/L	10–500 µg/L	[42]
			5% phenyl polysiloxane (15 m × 0.25 mm, 0.2 µm df)	n.r.	n.r.	25–10000 ng/mL	[116]
		GC/MS	HP-5MS (20 m × 0.25 mm, 0.25 µm film thickness)	1.7 ng/mL	5.7–5.8 ng/mL	5–500 µg/L	[117]
		HPLC-DAD	ODS-1 (150 × 4.6 mm) with precolumn (20 × 4.0 mm)	n.r.	7 ng/mL	n.r.	[121]
**MDA**	Plasma	HPLC-DAD	ODS-1 (150 × 4.6 mm) with precolumn (20 × 4.0 mm)	n.r.	5 ng/mL	n.r.	[121]
	Blood	LC/MS/MS	Kinetex C18 column (100 × 2.1 mm, 2.6 μm film thickness)	n.r.	0.0025 µg/L	0.0025–0.25 µg/L (R, S)	[122]
	Hair	GC/NICI-MS	HP-5MS (30 m × 0.25 mm, 0.25 µm film thickness)	1.6 pg/mg (*R*) 1.3 pg/mg (*S*)	5.3 pg/mg (*R*) 4.3 pg/mg (*S*)	0.005–60 ng/mg (*R*) 0.004–60 ng/mg (*S*)	[49]
		GC/MS	5% phenyl-methylsilicone capillary column (17 m × 0.2 mm, 0.33 µm film thickness)	0.1 ng/mg	0.2 ng/mg	0.2–20 ng/mg	[69]
	Urine	GC/NICI-MS LC/HRMS	n.r. (chiral derivatization S-HFBPrCl) Chirex3012 (250 × 4.6 mm, 5 µm film thickness)	n.r.	n.r.	n.r.	[68]
		GC/EI-MS	HP-5MS (20 m × 0.25 mm, 0.25 µm film thickness)	n.r.	2 µg/L	2–100 µg/L	[42]
			5% phenyl polysiloxane (15 m × 0.25 mm, 0.2 µm df)	n.r.	n.r.	25–10000 ng/mL	[116]
		HPLC-DAD	ODS-1 (150 × 4.6 mm) with precolumn (20 × 4.0 mm)	n.r.	5 ng/mL	n.r.	[121]
**MDEA**	Whole blood	GC/EI-MS	HP-5MS (30 m × 0.25 mm, 0.25 µm film thickness)	n.r.	0.004 µg/g	0.004–3 µg/g	[67]
	Hair	GC/NICI-MS	HP-5MS (30 m × 0.25 mm, 0.25 µm film thickness)	2.7 pg/mg (*R*) 2.3 pg/mg (*S*)	8.9 pg/mg (*R*) 7.7 pg/mg (*S*)	0.009–60 ng/mg (*R*) 0.008–60 ng/mg (*S*)	[49]
		GC/MS	5% phenyl-methylsilicone capillary column (17 m × 0.2 mm i.d., 0.33 µm film thickness)	0.2 ng/mg	0.5 ng/mg	0.5–20 ng/mg	[69]
	Urine	GC/EI-MS	5% phenyl polysiloxane (15 m × 0.25 mm, 0.2 µm df)	n.r.	n.r.	25–5000 ng/mL	[116]
***p*OHMA**	Blood	LC/MS/MS	Kinetex C18 column (100 × 2.1 mm, 2.6 μm film thickness)	n.r.	0.0025 µg/L	0.0025–0.25 µg/L (*R*,* S*)	[122]
	Urine	GC/NICI-MS LC/HRMS	n.r. (chiral derivatization *S*-HFBPrCl) Chirex3012 (250 × 4.6 mm, 5 µm film thickness)	n.r.	n.r.	n.r.	[68]
		CE/ESI-MS	Uncoated fused silica capillary (50 µm, 100 cm)	0.02 µg/mL	n.r.	0.05–10 µg/mL	[33]
				0.05 µg/mL	n.r.	0.2–10 µg/mL (*S*)	[118]
		CE	PVA chemically modified diol capillary column (40 cm × 50 µm)	n.r.	n.r.	n.r.	[36]
**HMMA**	Blood	LC/MS/MS	Kinetex C18 column (100 × 2.1 mm, 2.6 μm film thickness)	n.r.	0.0025 µg/L	0.0025–0.25 µg/L (*R*, *S*)	[122]
**Methadone**	Plasma	LC/EI-MS/MS	Chiral-AGP (50 × 2.0 mm, 5 µm)	n.r.	2.5 ng/mL	0–500 ng/mL	[85]
		LC/MS	Chiral-AGP (100 × 4.0 mm, 5 µm); Chiral-AGP guard column (10 × 2.0 mm, 5 µm)	0.02 ng/mL	1 ng/mL	1–300 ng/mL	[83]
	Blood	LC/ESI-MS/MS	Chiral-AGP (100 × 4.0 mm, 5 µm); Chiral-AGP guard column (10 × 2.0 mm, 5 µm)	n.r.	n.r.	n.r.	[12]
		LC/MS	α-1-acid glycoprotein (100 × 4.0 mm, 5 µm) and a α-1-acid glycoprotein guard column (10 × 2.0 mm, 5 µm)	n.r.	0.02 mg/L	0.05–2.1 mg/L	[123]
	Blood Tissues	LC/MS/MS	Chiral-AGP column (150 × 3 mm, 5 µm)	0.65 ng/L (*R*) 0.49 ng/L (*S*)	2.40 ng/L (*R*) 1.82 ng/L (*S*)	50–1000 ng/L	[82]
	Urine	LC/EI-MS/MS	Chiral-AGP (50 × 2.0 mm, 5 µm)	n.r.	2.5 ng/mL	0–500 ng/mL ER (*R/S*): 1.42–2.96	[85]
	Liver	LC/EI-MS/MS	Chiral-AGP (50 × 2.0 mm, 5 µm)	n.r.	2.5 ng/mL	0–500 ng/mL	[85]
**EDDP**	Plasma	LC/MS	Chiral-AGP (100 × 4.0 mm, 5 µm); Chiral-AGP guard column (10 × 2.0 mm, 5 µm)	0.01 ng/mL	0.1 ng/mL	1–25 ng/mL	[83]
		LC/EI-MS/MS	Chiral-AGP (50 × 2.0 mm, 5 µm)	n.r.	2.5 ng/mL	0–500 ng/mL	[85]
	Blood	LC/ESI-MS/MS	Chiral-AGP (100 × 4.0 mm, 5 µm); Chiral-AGP guard column (10 × 2.0 mm, 5 µm)	n.r.	n.r.	n.r.	[12]
	Blood Tissues	LC/MS/MS	Chiral-AGP column (150 × 3 mm, 5 µm)	0.77 ng/L (*R*) 0.76 ng/L (*S*)	2.82 ng/L (*R*) 2.79 ng/L (*S*)	50–1000 ng/L	[82]
	Urine	LC/EI-MS/MS	Chiral-AGP (50 × 2.0 mm, 5 µm)	n.r.	2.5 ng/mL	0–500 ng/mL ER (R/S): 0.76–0.89	[85]
	Liver	LC/EI-MS/MS	Chiral-AGP (50 × 2.0 mm, 5 µm)	n.r.	2.5 ng/mL	0–500 ng/mL	[85]
**T**	Plasma	LC/ESI-MS/MS	Chiralpak AD (250 × 4.6 mm, 10 µm)	n.r.	0.2 ng/mL (total) 0.5 ng/mL (unbound)	0.2–600 ng/mL (total) 0.5–250 ng/mL (unbound)	[81]
		LC/APCI-MS/MS	Lux Cellulose-2 (150 × 4.6 mm, 3 µm); Lux Cellulose-2 guard column (4 × 3 mm)	0.15 ng/mL	1 ng/mL	1–800 ng/mL	[75]
			Chiralpak AD (250 × 4.6 mm, 10 µm)	1 ng/mL	3 ng/mL	25–1000 ng/mL	[34]
		HPLC-DAD	Chiralcel OD-R (250 × 4.6 mm, 10 µm); LiChrospher 60-RP-selected B (250 × 4 mm, 5 µm)	0.18 ng/mL (+) 0.16 ng/mL (*‒*)	1 ng/mL	1–500 ng/mL	[124]
		HPLC-FD	Chiralpak AD (250 × 4.6 mm, 10 µm)	1 nM	5 nM	0.01–1.55 µM	[125]
			Chiralpak AD (250 × 4.6 mm); LichroCART 4-4 LiChrospher 100 Diol (5 µm) precolumn	n.r.	2.5 ng/mL	2.5–250 ng/mL	[126]
			Chiral-AGP (150 × 4.0 mm, 5 µm); Chiral-AGP guard column (10 × 4.0 mm, 5 µm)	n.r.	2 ng/mL	2–200 ng/mL	[127]
	Urine	GC/EI-MS	Rt-βDEXcst (30m × 0.25 mm, 0.25 µm film thickness)	0.01 µg/mL	0.1 µg/mL	0.1–20 µg/mL	[128]
		HPLC-FD	Chiralpak AD (250 × 4.6 mm, 10 µm)	2 nM	25 nM	0.1–3.0 µM	[125]
**ODT**	Plasma	LC/ESI-MS/MS	Chiralpak AD (250 × 4.6 mm, 10 µm)	n.r.	0.1 ng/mL (total) 0.25 ng/mL (unbound)	0.1–300 ng/mL (total) 0.25–125 ng/mL (unbound)	[81]
		LC/APCI-MS/MS	Lux Cellulose-2 (150 × 4.6 mm, 3 µm); Lux Cellulose-2 guard column (4 × 3 mm)	0.20 ng/mL (+) 0.30 ng/mL (*‒*)	1 ng/mL	1–400 ng/mL	[75]
			Chiralpak AD (250 × 4.6 mm, 10 µm)	1.3 ng/mL	4 ng/mL	25–1000 ng/mL	[34]
		HPLC-DAD	Chiralcel OD-R (250 × 4.6 mm, 10 µm); LiChrospher 60-RP-selected B (250 × 4 mm, 5 µm)	0.08 ng/mL (+) 0.06 ng/mL (*‒*)	0.5 ng/mL	0.5–100 ng/mL	[124]
		HPLC-FD	Chiralpak AD (250 × 4.6 mm, 10 µm)	1 nM	5 nM	0.01–1.55 µM	[125]
			Chiralpak AD (250 × 4.6 mm); LichroCART 4-4 LiChrospher 100 Diol (5 µm) precolumn	n.r.	2.5 ng/mL	2.5–250 ng/mL	[126]
			Chiral-AGP (150 × 4.0 mm × 5 µm); Chiral-AGP guard column (10 × 4.0 mm × 5 µm)	n.r.	2.5 ng/mL	2.5–100 ng/mL	[127]
	Urine	GC/EI-MS	Rt-βDEXcst (30 m × 0.25 mm, 0.25 µm film thickness)	0.03 µg/mL	0.1 µg/mL	0.1–20 µg/mL	[128]
		HPLC-FD	Chiralpak AD (250 × 4.6 mm, 10 µm)	2 nM	25 nM	0.1–3.0 µM	[125]
**NDT**	Plasma	LC/ESI-MS/MS	Chiralpak AD (250 × 4.6 mm, 10 µm)	n.r.	0.1 ng/mL (total) 0.25 ng/mL (unbound)	0.1–300 ng/mL (total) 0.25–125 ng/mL (unbound)	[81]
		HPLC-DAD	Chiralcel OD-R (250 × 4.6 mm, 10 µm); LiChrospher 60-RP-selected B (250 × 4 mm, 5 µm)	0.15 ng/mL (+) 0.16 ng/mL (*‒*)	0.5 ng/mL	0.5–250 ng/mL	[124]
		HPLC-FD	Chiral-AGP (150 × 4.0 mm, 5 µm); Chiral-AGP guard column (10 × 4.0 mm, 5 µm)	n.r.	2.5 ng/mL	2.5–75 ng/mL	[127]
**N,O-DDM-T**	Plasma	HPLC-FD	Chiralpak AD (250 × 4.6 mm); LichroCART 4-4 LiChrospher 100 Diol (5 µm) precolumn	n.r.	2.5 ng/mL	2.5–250 ng/mL	[126]
**Citalopram**	Plasma	LC/ESI-MS/MS	Chiralcel OD-R (250 × 4.6 mm × 10 µm); LiChrospher 100 RP-8 precolumn (4 × 4.0 mm × 5 µm)	n.r.	0.1 ng/mL	0.1–20 ng/mL	[129]
**FLX**	Plasma	LC/APCI-MS/MS	Chirobiotic V (250 × 4.6 mm, 5 µm)	n.r.	2 ng/mL	2–1000 ng/mL	[93]
**K**	Plasma	LC/MS	Chiral-AGP (100 × 4.0 mm, 5 µm); Chiral-AGP guard column (10 × 2.0 mm, 5 µm)	0.25 ng/mL	1 ng/mL	1–125 ng/mL	[111]
	Hair	CE-UV-DAD	Uncoated fused-silica capillary (450 × 50 mm)	0.08 ng/mg	0.25 ng/mg	0.5–8.0 ng/mg	[110]
**NK**	Plasma	LC/MS	Chiral-AGP (100 × 4.0 mm, 5 µm); Chiral-AGP guard column (10 × 2.0 mm, 5 µm)	0.25 ng/mL	1 ng/mL	1–125 ng/mL	[111]
	Hair	CE-UV-DAD	Uncoated fused-silica capillary (450 × 50 mm)	0.08 ng/mg	0.25 ng/mg	0.5–8.0 ng/mg	[110]
**MPH**	Plasma	GC/NICI-MS	BPX5 fused silica (15 m × 0.25 mm)	n.r.	0.006 ng/mL	0.006–12.5 ng/mL	[130]
				n.r.	0.072 ng/mL	0.072–18.25 ng/mL	[131]
	Blood	LC/MS/MS	Chiral AGP (100 × 4.0 mm, 5 µm); guard column (10 × 2.0 mm, 5 µm)	n.r.	n.r.	0.2–500 ng/g	[65]
	Urine	GC/EI-MS	DB-5 (30 m × 0.32 mm, 0.25 µm film thickness)	n.r.	10 ng/mL	0–10000 ng/mL	[132]
**Reboxetine**	Serum	LC/MS	Chiral-AGP (2 × 100 mm, 5 µm)	<1 nmol/L	n.r.	50–500 nmol/L ER (*S/R*): 0.22–0.88, with a mean value of 0.5	[97]
**VNF**	Plasma	LC/ESI-MS/MS	Chirobiotic V (250 × 2.1 mm, 5 µm)	n.r.	0.5 nM	1–1000 nM ER (*S/R*): 1.01–4.33	[95]
		HPLC/ESI-MS	Chirobiotic V (250 × 4.6 mm, 5 µm)	1 ng/mL	5.2 ng/mL (*R*) 5 ng/mL (*S*)	5–400 ng/mL	[133]
	Whole blood	LC/ESI-MS/MS	Chirobiotic V (250 × 2.1 mm, 5 µm)	n.r.	0.5 nM	10–4000 nM ER (*S/R*): 0.59–1.11	[95]
**OD-VNF**	Plasma	LC/ESI-MS/MS	Chirobiotic V (250 × 2.1 mm, 5 µm)	n.r.	0.5 nM	1–1000 nM ER (*S/R*): 0.70–12.3	[95]
		HPLC/ESI-MS	Chirobiotic V (250 × 4.6 mm, 5 µm)	1.5 ng/mL	3.5 ng/mL (*R*) 4.3 ng/mL (*S*)	4–280 ng/mL	[133]
	Whole blood	LC/ESI-MS/MS	Chirobiotic V (250 × 2.1 mm, 5 µm)	n.r.	0.5 nM	10–4000 nM ER (*S/R*): 0.59–1.11	[95]
**ND-VNF**	Plasma	LC/ESI-MS/MS	Chirobiotic V (250 × 2.1 mm, 5 µm)	n.r.	0.25 nM	0.5–500 nM ER (*S/R*): 1.24–2.91	[95]
	Whole blood	LC/ESI-MS/MS	Chirobiotic V (250 × 2.1 mm, 5 µm)	n.r.	0.25 nM	5–2000 nM ER (S/R): 0.46–1.53	[95]
**N,O-DD-VNF**	Plasma	LC/ESI-MS/MS	Chirobiotic V (250 × 2.1 mm, 5 µm)	n.r.	0.25 nM	0.5–500 nM ER (*S/R*): 0.42–1.18	[95]
	Whole blood	LC/ESI-MS/MS	Chirobiotic V (250 × 2.1 mm, 5 µm)	n.r.	0.25 nM	5–2000 nM ER (*S/R*): 0.90–1.99	[95]
**MET**	Urine	GC/EI-MS	HP-5MS (30 m × 0.25 mm, 0.25 µm film thickness)	0.5 ng/mL	n.r.	0.1–4 ng/mL ER (*R/S*): 0.83	[100]
**PHO**	Plasma	LC/APCI-MS	Chirobiotic V (250 × 4.6 mm, 5 µm); Chirobiotic V guard column (20 × 4.0 mm, 5 µm)	0.03 ng/mL	0.25 ng/mL	0.25–200 ng/mL	[98]
**WFN**	Plasma	SFC/APCI-MS/MS	Chiralpak AD (250 × 4.6 mm); Chiralpak AD-H guard column (10 × 4.0 mm)	n.r.	13.6 ng/mL	13.6–2500 ng/mL	[104]
		LC/ESI-MS/MS	Chirobiotic V (250 × 4.6 mm, 5 µm); Cyclobond I guard column (20 × 4.0 mm, 5 µm)	1.5 ng/mL	5 ng/mL	5–1500 ng/mL ER (*S/R*): 0.47±0.14	[105]
		MEKC/ESI-MS	Fused silica capillaries (120 cm, 375 µm o.d., 50 µm)	0.1 µg/mL (instr. limit)	n.r.	0.25–5 µg/mL ER (*R/S*): 2.24–6.20	[106]
**Carvedilol**	Plasma	LC/ESI-MS/MS	Chirobiotic T (250 × 4.6 mm, 10 µm)	n.r.	0.2 ng/mL	0.2–500 ng/mL	[99]
			Ace 3 C_18_ (50 × 2.0 mm, 3 µm)	n.r.	0.2 ng/mL	0.2–200 ng/mL	[134]
**Verapamil**	Plasma	CE-UV	Fused-silica capillaries	n.r.	n.r.	2.5–250 ng/mL	[101]
**Norverapamil**	Plasma	CE-UV	Fused-silica capillaries	n.r.	n.r.	2.5–250 ng/mL	[101]
**SBT**	Urine	NACE/ESI-MS	Fused silica capillaries (48.5 cm, 375 µm o.d., 50 µm)	8–14 ng/mL	18–20 ng/mL	15–150 ng/mL	[135]
**PTHIT**	Urine	GC-FID	Rt-β-DEXsm (30 m × 0.25 mm, 0.25 µm film thickness	n.r.	n.r.	n.r.	[10]

AM: amphetamine; CE: capillary electrophoresis; CI: chemical ionization; DAD: diode array detection; EDDP: 2-ethylidene-l,5-dimethyl-3,3-diphenylpyrrolidine; EI: electron impact; ER: enantiomeric ratio; ESI: electrospray ionization; FD: fluorescence detector; FID: flame ionization detector; FLX: fluoxetin; GC: gas chromatography; HMMA: 4-hydroxy-3-methoxymethamphetamine; HPLC: high performance liquid chromatography; K: ketamine; LC: liquid chromatography; LOD: limit of detection; LOQ: limit of quantification; MA: methamphetamine; MDA: 3,4-Methylenedioxyamphetamine; MDMA: 3,4 Methylenedioxymethamphetamine; MDEA: *N*-methyl-diethanolamine; MEKC: micellar electrokinetic chromatography; MET: metoprolol; MPH: methylphenidate; MS: mass spectrometry; MS/MS: tandem mass spectrometry; NACE: nonaqueous capillary electrophoresis; NDT: *N*-desmetil-tramadol; ND-VNF: *N*-desmethylvenlafaxine; NICI: negative ion chemical ionization; N,O-DD-VNF: *N*,*O*-didesmethylvenlafaxine; NFLX: norfluoxetin; NK: norketamine; ODT: *O*-desmetil-tramadol; OD-VNF: *O*-desmetil-venlafaxine; PHO: propranolol; PTHIT: phenyltetrahydroimidazothiazole; SBT: salbutamol; T: tramadol; UV: ultraviolet detector; VNF: venlafaxine; WFN: warfarin. n.r.: not referred.

**Table 3:** Analytical methods of separation of several chiral illicit drugs and pharmaceuticals in different environmental matrices.

Drugs	Matrix Application	Method	Stationary Phase	LOD/MDL	LOQ/MQL	Concentration Range/EF	Reference
**AM** **MA** **MDMA** **MDA** **Ephedrine**	Wastewater	UPLC/ESI-MS/MS	Chiral-CBH (100 × 2 mm, 5 µm); Chiral-CBH guard column (10 × 2 mm)	n.r.	AM: 5.1 ng/L	0.5–1000 ng/L; EF: 0.52–0.84 (mean 0.64)	[17]
					MA: 0.6 ng/L	0.05–1000 ng/L; EF ≥ 0.5	
					MDMA: 0.7 ng/L	0.1–1000 ng/L; EF = 0.68	
					MDA: 4.2 ng/L	0.1–1000 ng/L; EF > 0.5	
					Ephedrine: 5.6 ng/L	0.5–1000 ng/L; EF: 0.81–0.96 (mean 0.91)	
**AM, MA, MDMA, MDA, Ephedrine, Pseudoephedrine, Norephedrine, Atenolol, Alprenolol, PHO, MET, T, SBT, Sotalol, FLX, Mirtazapine, VNF, OD-VNF, Citalopram, *D*-citalopram**	Influent wastewater (IW); effluent wastewater (EW); digested sludge (DS)	LC/ESI-MS/MS	Chiral-CBH (100 × 2 mm, 5 µm); Chiral-CBH guard column (10 × 2 mm)	AM (*R*/*S*): 0.38/0.39 ng/L (IW); 0.28/0.41 ng/L (EW); 4.92/5.15 ng/L (DS)	AM (*R*/*S*): 1.28/ 1.32 ng/L (IW); 0.94/1.36 ng/L (EW); 16.56/17.28 ng/L (DS)	0.025–250 µg/L; EF: 0.5 (IW); 0.6 (EW); 0.3 (DS)	[62]
				MA (*R*/*S*): 0.12/0.13 ng/L (IW); 0.09/0.08 ng/L (EW); 0.73/0.77 ng/L (DS)	MA (*R*/*S*): 0.38/0.41 ng/L (IW); 0.28/0.27 ng/L (EW); 3.24/2.45 ng/L (DS)	0.025–250 µg/L EF: 0.6 (IW); 0.5 (EW); 0.5 (DS)	
				MDMA: 0.05 ng/L (IW); 0.04 ng/L (EW); 1.43/1.79 ng/L (*R*/*S*) (DS)	MDMA (*R*/*S*): 0.17/0.18 ng/L (IW); 0.13/0.14 ng/L (EW); 4.75/5.96 ng/L (DS)	0.025–250 µg/L EF: 0.7 (IW); 0.9 (EW); 0.4 (DS)	
				MDA (*R*/*S*): 0.33/0.36 ng/L (IW); 0.21/0.25 ng/L (EW); 2.21/4.14 ng/L (DS)	MDA (*R*/*S*): 1.13/1.19 ng/L (IW); 0.72/0.83 ng/L (EW); 7.46/13.74 ng/L (DS)	0.025–250 µg/L EF: 0.6 (IW); 0.5 (EW); 0.3 (DS)	
				Ephedrine (1*R*,2*S*/ 1*S*,2*R*): 0.23/0.16 ng/L (IW); 0.14/0.07 ng/L (EW)	Ephedrine (1*R*,2*S*/1*S*,2*R*): 0.01/0.02 ng/L (IW); 0.48/0.25 ng/L (EW)	0.025–250 µg/L EF: 0 (IW, EW)	
				Pseudoephedrine (1*R*,2*R*/1*S*,2*S*): 0.26/0.1 ng/L (IW); 0.15/0.11 ng/L (EW); 15.54/44.53 ng/L (DS)	Pseudoephedrine (1*R*,2*R*/1*S*,2*S*): 0.01/0.03 ng/L (IW); 0.52/0.36 ng/L (EW); 51.62/148.5 ng/L (DS)	0.025–250 µg/L EF: 1 (IW); 0.2 (EW)	
				Norephedrine (E1/E2): 0.33/0.15 ng/L (IW); 0.19/0.21 ng/L (EW); 9.54/13.05 ng/L (DS)	Norephedrine (E1/E2): 0.01/0.02 ng/L (IW); 0.63/0.71 ng/L (EW); 31.52/43.38 ng/L (DS)	0.025–250 µg/L EF: 0 (IW); 0.3 (EW); 0.1 (DS)	
			Chirobiotic V (250 × 2.1 mm, 5 µm)	Atenolol (*R*/*S*): 28.74/17.40 ng/L (IW); 30.80/32.73 ng/L (EW); 7.55/7.12 ng/L (DS)	Atenolol (*R*/*S*): 95.81/58 ng/L (IW); 102.68/109.08 ng/L (EW); 25.15/23.70 ng/L (DS)	0.025–250 µg/L EF: 0.5 (IW, EW); 0.4 (DS)	
				Alprenolol (*R*/*S*): 0.14/0.07 ng/L (IW); 0.06/0.03 ng/L (EW); 0.14 ng/L (DS)	Alprenolol (*R*/*S*): 0.47/0.24 ng/L (IW); 0.21/0.09 ng/L (EW); 0.48/0.46 ng/L (DS)	0.025–250 µg/L EF: 0.5 (IW, EW); 0.7 (DS)	
				PHO (*R*/*S*): 0.08/0.09 ng/L (IW); 0.06/0.05 ng/L (EW); 0.07/0.06 ng/L (DS)	PHO (*R*/*S*): 0.26/0.3 ng/L (IW); 0.20/0.17 ng/L (EW); 0.23/0.20 ng/L (DS)	0.025–250 µg/L EF: 0.4 (IW, EW); 0.5 (DS)	
				MET (*R*/*S*): 0.08/0.06 ng/L (IW); 0.05/0.04 ng/L (EW); 0.05/0.07 ng/L (DS)	MET (*R*/*S*): 0.27/0.18 ng/L (IW); 0.15/0.12 ng/L (EW); 0.15/0.22 ng/L (DS)	0.025–250 µg/L EF: 0.3 (IW, DS)	
				T (E1/E2): 0.09/0.43 ng/L (IW); 0.05/0.24 ng/L (EW); 0.05/0.34 ng/L (DS)	T (E1/E2): 0.29/1.43 ng/L (IW); 0.16/0.79 ng/L (EW); 0.18/1.13 ng/L (DS)	0.025–250 µg/L EF: 0.7 (IW, EW, DS)	
				SBT (*R*/*S*): 2.22/2.20 ng/L (IW); 1.31/0.98 ng/L (EW); 80.03/65.03 ng/L (DS)	SBT (*R*/*S*): 7.41/7.32 ng/L (IW); 4.36/3.26 ng/L (EW); 265.10/225.10 ng/L (DS)	0.025–250 µg/L EF: 0.5 (IW, EW)	
				Sotalol (E1/E2): 0.66/0.61 ng/L (IW); 0.53/0.46 ng/L (EW); 1.64/0.76 ng/L (DS)	Sotalol (E1/E2): 2.20/2.05 ng/L (IW); 1.76/1.53 ng/L (EW); 5.47/5.87 ng/L (DS)	0.025–250 µg/L EF: 0.5 (IW, EW, DS)	
				FLX (*R*/*S*): 0.08/0.07 ng/L (IW); 0.05/0.04 ng/L (EW); 0.09/0.07 ng/L (DS)	FLX (*R*/*S*): 0.26/0.22 ng/L (IW); 0.17/0.14 ng/L (EW); 0.30/0.23 ng/L (DS)	0.025–250 µg/L EF: 0.7 (IW, EW, DS)	
				Mirtazapine (*R*/*S*): 0.40/1.17 ng/L (IW); 0.40/0.86 ng/L (EW); 0.31/0.73 ng/L (DS)	Mirtazapine (*R*/*S*): 1.32/3.89 ng/L (IW); 1.32/2.86 ng/L (EW); 1.02/2.44 ng/L (DS)	0.025–250 µg/L EF: 0.3 (IW); 0.2 (EW); 0.5 (DS)	
				VNF (*R*/*S*): 0.03/0.04 ng/L (IW); 0.02/0.03 ng/L (EW); 0.03 ng/L (DS)	VNF (*R*/*S*): 0.11/0.12 ng/L (IW); 0.07/0.11 ng/L (EW); 0.08 ng/L (DS)	0.025–250 µg/L EF: 0.5 (IW, EW, DS)	
				OD-VNF (*R*/*S*): 0.32/0.16 ng/L (IW); 0.38/0.08 ng/L (EW); 0.75/1.02 ng/L (DS)	OD-VNF (*R*/*S*): 1.05/3.85 ng/L (IW); 1.28/2.30 ng/L (EW); 2.51/3.41 ng/L (DS)	0.025–250 µg/L EF: 0.5 (IW, EW, DS)	
				Citalopram (*R*/*S*): 0.31/0.24 ng/L (IW); 0.27/0.21 ng/L (EW); 0.21/0.09 ng/L (DS)	Citalopram (*R*/*S*): 13.69/13.07 ng/L (IW); 11.78/11.15 ng/L (EW); 9.09/4.69 ng/L (DS)	0.025–250 µg/L EF: 0.6 (IW, DS); 0.7 (EW)	
				*D*-Citalopram (*R*/*S*): 0.50/0.36 ng/L (IW); 0.40/0.29 ng/L (EW); 0.42/0.36 ng/L (DS)	D-Citalopram (*R*/*S*): 1.68/1.21 ng/L (IW); 1.34/0.96 ng/L (EW); 1.99/1.22 ng/L (DS)	0.025–250 µg/L EF: 1 (IW); 0.6 (DS)	
**AM, MA, MDMA, MDA, MDEA, Norephedrine, VNF**	WWTP influent (IW); WWTP effluent (EW)	UPLC/ESI-MS/MS	Chiral-CBH (100 × 2 mm, 5 µm); Chiral-CBH guard column (10 × 2 mm)	AM (*R*/*S*): 0.85/0. 9 ng/L (IW); 0.9/0.85 ng/L (EW)	AM (*R*/*S*): 4.35/4.4 ng/L (IW); 4.4/4.35 ng/L (EW)	0.25–1900 ng/L EF n.r.	[149]
				MA: 0.85/0. 9 ng/L (*R*/*S*) (IW); 1 ng/L (EW)	MA: 2.8/2.95 ng/L (*R*/*S*) (IW); 3.35 ng/L (EW)	0.25–1900 ng/L EF n.r.	
				MDMA: 0.9 ng/L (IW); 0.95/1 ng/L (E1/E2) (EW)	MDMA: 2.4 ng/L (IW); 2.55–2.65 ng/L (E1/E2) (EW)	0.25–1900 ng/L EF: 0.53–0.72 (mean 0.63) (IW); 0.71 (EW)	
				MDA: 1.95/2 ng/L (E1/E2) (IW); 2 ng/L (EW)	MDA: 9.7 ng/L (IW); 10.1 ng/L (EW)	0.25–1900 ng/L EF n.r.	
				MDEA: 0.55 ng/L (IW); 0.6 ng/L (EW)	MDEA: 2.25/2.2 ng/L (E1/E2) (IW); 2.4 ng/L (EW)	0.25–1900 ng/L EF n.r.	
				Norephedrine (E1/E2): 3.5/3.3 ng/L (IW); 1.4/1.35 ng/L (EW)	Norephedrine (E1/E2): 11.75/10.9 ng/L (IW); 4.6/4.5 ng/L (EW)	0.25–1900 ng/L EF n.r.	
				VNF: 1.6 ng/L (IW); 1.65 ng/L (EW)	VNF: 4.95/5.05 ng/L (E1/E2) (IW); 5.1 ng/L (EW)	0.25–1900 ng/L EF: 0.45–0.50 (mean 0.48) (IW); 0.37–0.48 (mean 0.43) (EW)	
**AM, MA, MDMA, MDA, Ephedrine, Atenolol, VNF**	WWTP influent (IW); WWTP effluent (EW); river water (RW)	LC/ESI-MS/MS	Chiral-CBH (100 × 2 mm, 5 µm); Chiral-CBH guard column (10 × 2 mm)	n.r.	n.r.	AM: 1–500 ng/L EF: 0.52–0.84 (mean 0.64) (IW); 0.57–1 (mean 0.78) (EW); 0.86 (RW before WWTP); 0.81 (RW after WWTP)	[9]
						MA: 1–500 ng/L EF: 0.22–0.53 (mean 0.34) (IW); 0.7–1 (mean 0.86) (EW)	
						MDMA: 1–500 ng/L EF: 0.5–0.8 (mean 0.66) (IW); 0.64–0.91 (mean 0.75) (EW); 0.56–0.81 (mean 0.68) (RW before WWTP); 0.61–0.80 (mean 0.69) (RW after WWTP)	
						MDA: 1–500 ng/L EF: 0.26–0.47 (mean 0.34) (IW); 0.38–0.58 (mean 0.45) (EW); 0.58 (RW before WWTP); 0.56–0.57 (RW after WWTP)	
						Ephedrine: 1–500 ng/L EF: 0.81–1 (mean 0.99) (IW); 0.22–1 (mean 0.92) (EW); 0.79–1 (mean 0.97) (RW before WWTP); 0.80–1 (mean 0.99) (RW after WWTP) DF: 0.02–0.66 (mean 0.26) (IW); 0.04–0.82 (mean 0.36) (EW); 0–1 (mean 0.6) (RW before WWTP); 0–1 (mean 0.46) (RW after WWTP)	
						VNF: 1–500 ng/L EF: 0.35–0.65 (mean 0.48) (IW); 0.46–0.69 (mean 0.52) (EW); 0.40–0.65 (mean 0.52) (RW before WWTP); 0.47–0.62 (mean 0.51) (RW after WWTP)	
					Atenolol: 1.7 ng/L (IW, EW); 0.3 ng/L (RW)	1–500 ng/L EF: 0.30–0.47 (mean 0.40) (IW); 0.40–0.61 (mean 0.46) (EW); 0.38–0.56 (mean 0.46) (RW before WWTP); 0.39–0.50 (mean 0.45) (RW after WWTP)	
**AM, MA, MDMA, MDA, Atenolol, PHO, MET, FLX, VNF**	Sewage effluent (SE); River water (RW)	LC/QTOF-MS	Chirobiotic V (250 × 4.6 mm, 5 µm); Chirobiotic V guard column (20 × 40 mm, 5 µm)	AM: 4.6/4.4 ng/L (*R*/*S*) (SE); 1.8 ng/L (RW)	AM (*R*/*S*): 12.4/11.5 ng/L (SE); 5.0/4.8 ng/L (RW)	0.5–500 ng/L EF n.r.	[151]
				MA (*R*/*S*): 11.9/14.2 ng/L (SE); 4.6/5.5 ng/L (RW)	MA (*R*/*S*): 47.6/47.3 ng/L (SE); 18.5/18.3 ng/L (RW)	0.25–500 ng/L EF n.r.	
				MDMA (E1/E2): 22.8/21.8 ng/L (SE); 9.6/10.4 ng/L (RW)	MDMA (E1/E2): 85.7/81.9 ng/L (SE); 35.8/39 ng/L (RW)	5–500 ng/L EF n.r.	
				Atenolol (*R*/*S*): 5/5.3 ng/L (SE); 2.1/2.2 ng/L (RW)	Atenolol (*R*/*S*): 11.4/11 ng/L (SE); 4.8/4.7 ng/L (RW)	0.25–100/5–500 ng/L (*R*); 0.5–100/5–500 ng/L (*S*) EF: 0.55 (SE); 0.47 (RW)	
				PHO (*R*/*S*): 1.4/1 ng/L (SE); 0.6/0.4 ng/L (RW)	PHO (R/S): 3.4/2.6 ng/L (SE); 1.4/1.2 ng/L (RW)	0.25–100/5–500 ng/L EF: 0.43 (SE); 0.45 (RW)	
				MET: 0.6/0.7 ng/L (E1/E2) (SE); 0.2 ng/L (RW)	MET: 1.3 ng/L (SE); 0.3/0.4 ng/L (E1/E2) (RW)	0.25–100/5–500 ng/L EF: 0.54 (SE)	
				FLX: 2.4/2.6 ng/L (*R*/*S*) (SE); 0.8 ng/L (RW)	FLX (*R*/*S*): 7.6/6.5 ng/L (SE); 2.5/2 ng/L (RW)	0.25–100/5–500 ng/L EF n.r.	
				VNF (*R*/*S*): 4.8/3.9 ng/L (SE); 2.5/2.2 ng/L (RW)	VNF (*R*/*S*): 15.1/14.4 ng/L (SE); 7.9/8.1 ng/L (RW)	0.5–100/5–500 ng/L EF: 0.43 (SE); 0.58 (RW)	
			Chiral-CBH (100 × 2 mm, 5 µm); Chiral-CBH guard column (10 × 2 mm, 5 µm)	AM (*R*/*S*): 4.8/5 ng/L (RW)	AM (*R*/*S*): 9.7/10 ng/L (RW)	0.5–500 ng/L EF n.r.	
				MA (*R*/*S*): 4.1/3.6 ng/L (RW)	AM (*R*/*S*): 20.6/18.1 ng/L (RW)	2.5–500 ng/L EF n.r.	
				MDMA (*R*/*S*): 10.7/10.2 ng/L (RW)	MDMA (*R*/*S*): 26.8/25.6 ng/L (RW)	12.5–500 ng/L EF n.r.	
				MDA (*R*/*S*): 2.4/2.3 ng/L (RW)	MDA (*R*/*S*): 9.6/9.1 ng/L (RW)	1.75–500 ng/L EF n.r.	
				Atenolol (*R*/*S*): 2.3/2.1 ng/L (RW)	Atenolol (*R*/*S*): 22.9/20.7 ng/L (RW)	0.5–500 ng/L EF n.r.	
				VNF (E1/E2): 10.3/9.6 ng/L (RW)	VNF (E1/E2): 51.7/47.9 ng/L (RW)	5–500 ng/L EF n.r.	
**Atenolol, PHO, MET, SBT, Sotalol, Nadolol, Pindolol, FLX, Citalopram**	Influent wastewater (IW); Effluent wastewater (EW)	LC/ESI-MS/MS	Chirobiotic V (250 × 4.6 mm, 5 µm) with a nitrile guard cartridge (10 × 3 mm)	Atenolol: 1.8 ng/L (IW); 1.4 ng/L (EW)	6 ng/L (IW); 5 ng/L (EW)	1–500 ng/mL EF n.r.	[152]
				PHO: 0.5 ng/L (IW, EW)	2 ng/L (IW, EW)		
				MET: 2.3 ng/L (IW); 0.6 ng/L (EW)	8 ng/L (IW); 2 ng/L (EW)		
				SBT: 0.7 ng/L (IW); 0.6 ng/L (EW)	2 ng/L (IW, EW)		
				Sotalol: 7.5 ng/L (IW); 7.2 ng/L (EW)	25 ng/L (IW); 24 ng/L (EW)		
				Nadolol: 1.8 ng/L (IW); 1.7 ng/L (EW)	12 ng/L (IW); 3 ng/L (EW)		
				Pindolol: 0.4 ng/L (IW); 0.2 ng/L (EW)	1 ng/L (IW, EW)		
				FLX: 2.2 ng/L (IW); 0.6 ng/L (EW)	7 ng/L (IW); 2 ng/L (EW)		
				Citalopram: 2.4 ng/L (IW); 0.5 ng/L (EW)	8 ng/L (IW); 2 ng/L (EW)		
**Atenolol, PHO, MET**	WWTP influent (IW); WWTP effluent (EW)	HPLC/ESI-MS/MS	Chirobiotic V (250 × 4.6 mm, 5 µm) with a nitrile guard cartridge (10 × 3 mm) and an in-line filter	Atenolol: 110 ng/L IW); 12 ng/L (EW)	n.r.	25–1000 ng/mL EF ≈ 0.5 (IW; EW)	[153]
				PHO: 17 ng/L (IW); 4.4 ng/L (EW)	n.r.	25–1000 ng/mL EF ≈ 0.5 (IW; EW)	
				MET: 42 ng/L (IW); 17 ng/L (EW)	n.r.	25–1000 ng/mL EF: 0.5 (IW); ≠0.5 (EW)	
**Atenolol, MET, Sotalol, Citalopram, Temazepam**	Effluent wastewater	LC/ESI-MS/MS	Chirobiotic V (250 × 4.6 mm, 5 µm)	n.r.	n.r.	Atenolol EF: 0.40–0.52 (mean 0.46)	[142]
						MET EF: 0.39–0.52 (mean 0.46)	
						Sotalol EF: 0.34–0.41 (mean 0.36)	
						Citalopram EF: 0.44–0.62 (mean 0.58)	
			Chiralpak AD-RH (150 × 4.6 mm, 5 µm)	n.r.	n.r.	Temazepam EF: 0.39–0.49 (mean 0.47)	
**Atenolol, PHO, MET, Bisoprolol**	River water	LC-UV	Lux Cellulose-1 (250 × 4.6 mm, 5 µm)	Atenolol: 22 µg/L	70 µg/L	12.5–100 µg/mL EF n.r.	[154]
				PHO: 3 µg/L	10 µg/L		
				MET: 20 µg/L	40 µg/L		
				Bisoprolol: 3 µg/L	10 µg/L		
**Alprenolol, PHO, MET, SBT, Bisoprolol, FLX, NFLX, VNF**	WWTP effluent	LC/ESI-MS/MS	Chirobiotic V (150 × 2.1 mm, 5 µm)	Alprenolol (*R*/*S*): 8.08/4.52 ng/L	Alprenolol (*R*/*S*): 18.5/13.7 ng/L	20–400 ng/L EF n.r.	[39]
				PHO (*R*/*S*): 1.97/0.65 ng/L	PHO (*R*/*S*): 5.96/1.98 ng/L		
				MET (*R*/*S*): 11.5/3.37 ng/L	MET (*R*/*S*): 14.8/10.2 ng/L		
				SBT (*R*/*S*): 5.07/6.29 ng/L	SBT (*R*/*S*): 15.4/19.1 ng/L		
				Bisoprolol (E1/E2): 2.78/4.54 ng/L	Bisoprolol (E1/E2): 8.44/13.8 ng/L		
				FLX (*R*/*S*): 8.41/3.74 ng/L	FLX (*R*/*S*):19.5/11.3 ng/L		
				NFLX (*R*/*S*): 0.97/5.27 ng/L	NFLX (*R*/*S*): 2.95/16 ng/L	30–400 ng/L EF n.r.	
				VNF (*R*/*S*): 9.82/1.71 ng/L	VNF (*R*/*S*): 19.7/5.18 ng/L	20–400 ng/L EF: 0.54–0.55 (mean 0.55)	
**Alprenolol, PHO, MET, FLX, VNF, Ibuprofen, Naproxen, Flurbiprofen**	Surface water	LC/ESI-MS/MS	Chirobiotic V (250 × 4.6 mm, 5 µm); Chirobiotic V guard column (20 × 4 mm, 5 µm)	Alprenolol (*R*/*S*): 0.2/0.1 ng/L	Alprenolol (*R*/*S*): 0.5/0.4 ng/L	5–1000 µg/L EF n.r.	[37]
				PHO (*R*/*S*): 0.6/0.5 ng/L	PHO (*R*/*S*): 2.1/1.7 ng/L	5–1000 µg/L EF: 0.44–0.56 (mean 0.49)	
				MET: 0.2 ng/L	MET (*R*/*S*): 0.6/0.5 ng/L	5–1000 µg/L EF: 0.48–0.64 (mean 0.55)	
				FLX: 0.1 ng/L	0.5 ng/L	5–1000 µg/L; EF: 0.5–0.63	
				VNF: 0.1 ng/L	0.5 ng/L	5–1000 µg/L; EF: 0.46–0.51 (mean 0.49)	
			Chiralpak AD-RH (150 × 4.6 mm, 5 µm)	Ibuprofen (*R*/*S*): 11/9.6 ng/L	Ibuprofen (*R*/*S*): 37/32 ng/L	5–1000 µg/L EF n.r.	
				Naproxen: 0.4 ng/L	Naproxen (*R*/*S*): 1.4/1.2 ng/L		
				Flurbiprofen (*R*/*S*): 3.3/2.4 ng/L	Flurbiprofen (*R*/*S*): 11/7.9 ng/L		
**PHO**	Influent wastewater (IW); Effluent wastewater (EW); Surface water (SW)	GC/ESI-MS/MS	MDN-5S (30 m × 0.25 mm, 0.25 µm film thickness)	n.r.	n.r.	EF: 0.5 (IW); ≤0.42 (EW); 0.42–0.53 (SW)	[155]
**PHO**	River water	LC-UV	Lux-Cellulose 1 (250 × 4.6 mm, 5 µm)	0.4 µg/L	1.3 µg/L	0.125–50 µg/mL; EF n.r.	[156]
**MET**	Influent wastewater (IW); Effluent wastewater (EW)	LC/ESI-MS/MS	Chirobiotic V (250 × 4.6 mm, 5 µm)	3.7/3.5 ng/L (IW); 1.9/1.5 ng/L (EW) (*R*/*S*)	12.4/11.5 ng/L (IW); 6.5/5.1 ng/L (EW) (*R*/*S*)	EF: 0.48–0.52 (IW); 0.5–0.7 (EW)	[40]
**MET**	Treated wastewater	LC/MS/MS	Chiral-CBH (100 × 2 mm, 5 µm); Chiral-CBH guard column and in-line high-pressure filter (4 mm, 0.5 µm)	0.96/2.9 pM (E1/E2)	5.8/11.6 pM (E1/E2)	EF: 0.51–0.55	[157]
**MET**	Effluent wastewater (EW); River water (RW)	GC/ESI-MS/MS	MDN-5S (30 m × 0.25 mm, 0.25 µm film thickness)	n.r.	n.r.	EF: 0.5 (EW); 0.31–0.44 (RW)	[158]
**FLX, NFLX**	Raw wastewater (RaW); Treated wastewater (TW)	LC/ESI-MS/MS	Chiral-AGP (100 × 2 mm, 5 µm); in-line high-pressure filter with a replaceable cap frit (4 mm, 5 µm); Chiral-AGP guard column (10 × 2 mm)	FLX: 3 pM (RaW); 2/1 pM (*R*/*S*) (TW)	FLX: 12.4 pM (RaW); 3 pM (TW)	0–500 pM EF: 0.71 (RaW, TW)	[159,160]
				NFLX: 2.4 pM (RaW); 2 pM (TW)	NFLX: 12.1/14.3 pM (E1/E2) (RaW); 4 pM (TW)	0–500 pM EF: 0.69 (RaW); 0.68 (TW)	
**FLX, NFLX**	WWTP effluent	HPLC-FD	Chirobiotic V (150 × 4.6 mm, 5 µm)	FLX: 0.8–2 ng/mL	4 ng/mL	4–60 ng/mL; EF n.r.	[137]
				NFLX: 0.8–2 ng/mL	2 ng/mL	2–30 ng/mL; EF n.r.	
**VNF**	River water	LC/ESI-MS/MS	Chirobiotic V (250 × 2.1 mm, 5 µm); Chirobiotic guard column (10 × 2 mm)	6/4 ng/L (*R*/*S*)	n.r.	EF: 0.46–0.74	[144]
**Ibuprofen, Carboxyibuprofen, 2-Hydroxyibuprofen, Naproxen, Ketoprofen, Indoprofen, Chloramphenicol, Ifosfamide, Praziquantel**	Influent wastewater (IW); Effluent wastewater (EW); Surface water (SW)	LC/ESI-MS/MS	Chirobiotic T (250 × 2.1 mm, 5 µm)	Ibuprofen (*R*/*S*): 1319/1111 ng/L (IW); 498/383 ng/L (EW); 263/114 ng/L (SW)	Ibuprofen (*R*/*S*): 5403/4551 ng/L (IW); 2039/1570 ng/L (EW); 1076/466 ng/L (SW)	250–400 µg/L EF: 1 (IW)	[161]
				Carboxyibuprofen (E1/E2): 71/63.6 ng/L (IW); 71.4/58.6 ng/L (EW); 21.5/22.3 ng/L (SW)	Carboxyibuprofen (E1/E2): 232/208 ng/L (IW); 233/191 ng/L (EW); 70.2/72.7 ng/L (SW)	32.7–300 µg/L (IW); 250–400 µg/L (EW, SW) EF: 0.83 (IW)	
				2-Hydroxyibuprofen (E1/E2): 31.7/20.4 ng/L (IW); 28/30.4 ng/L (EW); 10.9/10.4 ng/L (SW)	2-Hydroxyibuprofen (E1/E2): 104/66.4 ng/L (IW); 91.3/99.3 ng/L (EW); 35.4/33.9 ng/L (SW)	16.3–400 µg/L (E1); 16.3–300 µg/L (E2) EF: 0.76 (IW)	
				Naproxen (*R*/*S*): 11/7.53 ng/L (IW); 14.4/13.4 ng/L (EW); 7.5/6.83 ng/L (SW)	Naproxen (*R*/*S*): 38.1/26.1 ng/L (IW); 49.9/46.5 ng/L (EW); 25.9/23.7 ng/L (SW)	8.66–50 µg/L EF: 1 (IW)	
				Ketoprofen (*R*/*S*): 2.08/2.61 ng/L (IW); 2.28/2.56 ng/L (EW); 1.60/1.32 ng/L (SW)	Ketoprofen (*R*/*S*): 6.85/8.59 ng/L (IW); 7.51/8.44 ng/L (EW); 5.29/4.37 ng/L (SW)	1.65–400 µg/L EF n.r.	
				Indoprofen (E1/E2): 2.23/3.44 ng/L (IW); 2.20/2.59 ng/L (EW); 1.54/1.46 ng/L (SW)	Indoprofen (E1/E2): 7.59/11.7 ng/L (IW); 7.47/8.81 ng/L (EW); 5.24/4.95 ng/L (SW)	1.70–100 µg/L EF n.r.	
				Chloramphenicol (1*R,2R*/1S,2*S*): 29.1/5.66 ng/L (IW); 26.1/4.84 ng/L (EW); 13.5/2.59 ng/L (SW)	Chloramphenicol (1*R,2R*/1S,2*S*): 98.9/18.8 ng/L (IW); 88.6/16.1 ng/L (EW); 45.8/8.61 ng/L (SW)	17–400 µg/L (1*R,2R*); 3.33–800 µg/L (1*S*,2*S*) EF n.r.	
				Ifosfamide (E1/E2): 0.24/0.28 ng/L (IW); 0.23/0.22 ng/L (EW); 0.12/0.13 ng/L (SW)	Ifosfamide (E1/E2): 0.82/0.96 ng/L (IW); 0.78/0.74 ng/L (EW); 0.41/0.44 ng/L (SW)	0.17–50 µg/L EF n.r.	
				Praziquantel (E1/E2): 3.02/3.11 ng/L (IW); 2.78/2.82 ng/L (EW); 1.34/1.39 ng/L (SW)	Praziquantel (E1/E2): 10.1/10.4 ng/L (IW); 9.26/9.40 ng/L (EW); 4.47/4.63 ng/L (SW)	1.67–400 µg/L EF n.r.	
**Ibuprofen, Naproxen**	Influent wastewater (IW); Effluent wastewater (EW)	GC/MS	Astec Chiraldex (20 m × 0.25 mm, 0.12 µm film thickness)	0.1 µg/L	n.r.	Ibuprofen EF: 0.73–0.90 (IW); 0.60–0.76 (EW)	[44]
						Naproxen EF: 0.88–0.90 (IW); 0.71–0.86 (EW)	
**Ibuprofen, Naproxen, Ketoprofen**	Influent wastewater (IW); Effluent wastewater (EW)	GC/EI-MS/MS	HP5-MS (30 m × 0.25 mm, 0.25 µm film thickness)	n.r.	n.r.	Ibuprofen EF: 0.88–0.94 (IW); 0.38–0.40 (EW)	[162]
						Naproxen EF: 0.99 (IW); 0.86–0.94 (EW)	
						Ketoprofen EF: 0.56–0.60 (IW); 0.54–0.68 (EW)	
**Ibuprofen, 2-Hydroxyibuprofen, Naproxen, Indoprofen, Carprofen, Fenoprofen, Flurbiprofen, Chloramphenicol, Aminorex, Tetramisole, Omeprazole, Ifosfamide, 3-*N*-Dechloroethylifosfamide, Praziquantel, Imazalil, Ofloxacin**	Influent wastewater (IW); Effluent wastewater (EW)	UHPSFC/ESI-MS/MS	Polysaccharide amylose *tris*-(3,5-dimethylphenylcarbamate) column	Ibuprofen (*R*/*S*): 1410/1525 ng/L (IW); 1458/1452 ng/L (EW)	Ibuprofen (*R*/*S*): 4695/5080 ng/L (IW); 4854/4837 ng/L (EW)	415–2000 µg/L EF: 1 (IW)	[163]
				2-Hydroxyibuprofen (E1/E2): 409/415 ng/L (IW)	2-Hydroxyibuprofen (E1/E2): 1360/1382 ng/L (IW)	163.5–2000 µg/L EF: 0.2 (IW)	
				Naproxen: 233/267 ng/L (*R*/*S*) (IW); 539 ng/L (*R*) (EW)	Naproxen: 777/891 ng/L (*R*/*S*) (IW); 1796 ng/L (*R*) (EW)	84.3–2000 µg/L EF: 1 (IW, EW)	
				Indoprofen (E1/E2): 2.38/2.68 ng/L (IW); 2.88/2.65 ng/L (EW)	Indoprofen (E1/E2): 7.91/8.91 ng/L (IW); 9.60/8.84 ng/L (EW)	0.85–250 µg/L (E1); 0.85–500 µg/L (E2) EF n.r.	
				Carprofen (E1/E2): 378/287 ng/L (IW); 584/705 ng/L (EW)	Carprofen (E1/E2): 1259/956 ng/L (IW); 1945/2347 ng/L (EW)	168–500 µg/L EF n.r.	
				Fenoprofen (E1/E2): 571/538 ng/L (IW); 499/489 ng/L (EW)	Fenoprofen (E1/E2): 1900/1793 ng/L (IW); 1660/1632 ng/L (EW)	171–4000 µg/L EF n.r.	
				Flurbiprofen: 331 ng/L (IW); 252/378 ng/L (E1/E2) (EW)	Flurbiprofen (E1/E2): 838/1101 ng/L (IW); 838/1259 ng/L (EW)	83.8–2000 µg/L EF n.r.	
				Chloramphenicol (1*R,2R*/1S,2*S*): 45.6/43.5 ng/L (IW); 53.4/50.1 ng/L (EW)	Chloramphenicol (1*R,2R*/1S,2*S*): 152/145 ng/L (IW); 178/167 ng/L (EW)	16.9–500 µg/L (1*R,2R*); 16.7–500 µg/L (1S,2*S*) EF n.r.	
				Aminorex (E1/E2): 1.82/2.57 ng/L (IW); 2.16/3.02 ng/L (EW)	Aminorex (E1/E2): 6.05/8.56 ng/L (IW); 7.20/10 ng/L (EW)	0.83–500 µg/L EF n.r.	
				Tetramisole (*R*/*S*): 2.54/2.94 ng/L (IW); 2.83/2.87 ng/L (EW)	Tetramisole (*R*/*S*): 8.46/9.79 ng/L (IW); 9.43/9.54 ng/L (EW)	0.83–500 µg/L EF n.r.	
				Omeprazole: 24.5 ng/L (IW, EW)	81.6 ng/L (IW, EW)	0.82–125 µg/L (E1); 0.82–250 µg/L (E2) EF n.r.	
				Ifosfamide (E1/E2): 0.51/0.58 ng/L (IW); 0.51/0.54 ng/L (EW)	Ifosfamide (E1/E2): 1.70/1.93 ng/L (IW); 1.69/1.78 ng/L (EW)	0.17–125 µg/L EF n.r.	
				3-*N*-Dechloroethyl-ifosfamide (E1/E2): 0.46/3.22 ng/L (IW); 1.35/8.62 ng/L (EW)	3-*N*-Dechloroethyl-ifosfamide (E1/E2): 1.54/10.70 ng/L (IW); 4.50/28.70 ng/L (EW)	0.17–50 µg/L (E1); 0.83–125 µg/L (E2) EF n.r.	
				Praziquantel (E1/E2): 2.66/2.47 ng/L (IW); 2.64/2.77 ng/L (EW)	Praziquantel (E1/E2): 8.86/8.23 ng/L (IW); 8.78/9.23 ng/L (EW)	0.83–50 µg/L EF n.r.	
			Cellulose *tris*-(3-chloro-4-methylphenylcarbamate) column	Indoprofen (E1/E2): 5.53/3.94 ng/L (IW); 6.82/5.52 ng/L (EW)	Indoprofen (E1/E2): 18.4/13.1 ng/L (IW); 22.7/18.4 ng/L (EW)	1.69–500 µg/L (E1); 1.69–250 µg/L (E2) EF n.r.	
				Aminorex (E1/E2): 2.32/2.54 ng/L (IW); 2.23/3.44 ng/L (EW)	Aminorex (E1/E2): 7.74/8.44 ng/L (IW); 7.59/11.70 ng/L (EW)	0.83–500 µg/L EF: 0.4 (IW)	
				Tetramisole (*R*/*S*): 2.72/3.08 ng/L (IW); 3.16/2.60 ng/L (EW)	Tetramisole (*R*/*S*): 9.06/10.30 ng/L (IW); 10.50/8.65 ng/L (EW)	0.83–250 µg/L (*R*); 0.83–500 µg/L (*S*) EF: 0.6 (IW, EW)	
				Omeprazole: 49 ng/L (IW, EW)	163 ng/L (IW, EW)	1.63–500 µg/L EF n.r.	
				3-*N*-Dechloroethyl-ifosfamide (E1/E2): 2.81/2.99 ng/L (IW); 7.69/8.68 ng/L (EW)	3-*N*-Dechloroethyl-ifosfamide (E1/E2): 9.35/9.97 ng/L (IW); 25.60/28.90 ng/L (EW)	0.83–125 µg/L EF: 0.4 (IW)	
				Praziquantel (E1/E2): 6.62/5.59 ng/L (IW); 6.80/5.30 ng/L (EW)	Praziquantel (E1/E2): 22/18.60 ng/L (IW); 22.60/17.60 ng/L (EW)	1.67–500 µg/L (E1); 1.67–250 µg/L (E2) EF n.r.	
				Imazalil (E1/E2): 5.12/5.16 ng/L (IW); 7.09/6.45 ng/L (EW)	Imazalil (E1/E2): 17/17.20 ng/L (IW); 23.60/21.50 ng/L (EW)	1.74–500 µg/L (E1); 1.74–250 µg/L (E2) EF: 0 (IW)	
				Ofloxacin (E1/E2): 98.20/63.10 ng/L (IW); 65.20/79 ng/L (EW)	Ofloxacin (E1/E2): 327/210 ng/L (IW); 218/263 ng/L (EW)	16.4–500 µg/L (E1); 16.4–250 µg/L (E2) EF: 0 (IW)	
**Ibuprofen, Naproxen, Ketoprofen**	Effluent wastewater	GC/EI-MS/MS	HP5-MS (30 m × 0.25 mm, 0.25 µm film thickness)	Ibuprofen: 0.7 ng/L (*S*)	n.r.	0.08–300 ng/L; EF: 0.49–0.62 (mean 0.53)	[147]
				Naproxen: 0.7 ng/L (*S*)	n.r.	0.08–300 ng/L; EF: 0.66–0.86 (mean 0.79)	
				Ketoprofen: 2.2 ng/L (*S*)	n.r.	3–300 ng/L; EF: 0.54–0.66 (mean 0.60)	
**Ibuprofen, Naproxen**	Influent wastewater (IW); Effluent wastewater (EW)	GC/EI-MS/MS	HP5-MS (30 m × 0.25 mm, 0.25 µm film thickness)	n.r.	n.r.	Ibuprofen EF: 0.6–0.8 (IW); 0.5 (EW)	[164]
						Naproxen EF: 1 (IW); 0.7–0.9 (EW)	
**Ibuprofen, Naproxen, Ketoprofen**	Influent wastewater (IW); Effluent wastewater (EW)	LC/MS/MS	Sumichiral OA-2500 (250 × 4.6 mm, 5 µm); Chirex 3005 guard column (30 × 4.6 mm, 5 µm)	Ibuprofen: 0.7 ng/L (IW); 0.5 ng/L (EW)	n.r.	0.4–4000 µg/L EF: 0.79–0.86 (IW); 0.63–0.68 (EW)	[165]
				Naproxen: 1.2/1.1 ng/L (*R*/*S*) (IW); 1.1 ng/L (EW)	n.r.	1.2–4000 µg/L EF: 0.98–0.99 (IW); 0.93–0.96 (EW)	
				Ketoprofen (*R*/*S*): 0.9/0.8 ng/L (IW); 0.8/0.7 ng/L (EW)	n.r.	1–4000 µg/L EF: 0.54–0.68 (IW); 0.61–0.68 (EW)	
**Ibuprofen, Naproxen, Ketoprofen, Chloramphenicol, Aminorex, Tetramisole, Ifosfamide, 3-*N*-Dechloroethylifosfamide, Fexofenadine, 10,11-Dihydro-10-hydroxy-carbamazepine, Praziquantel**	Effluent wastewater (EW); Surface water (SW)	LC/ESI-MS/MS	Chiral-AGP (100 × 2 mm, 5 µm); Chiral-AGP guard column (10 × 2 mm, 5 µm)	Ibuprofen (*R*/*S*): 16.45/23.15 ng/L (EW); 9.15/9.39 ng/L (SW)	Ibuprofen (*R*/*S*): 67.37/94.81 ng/L (EW); 37.47/38.46 ng/L (SW)	41–492 µg/L EF: 0.65 (EW)	[32]
				Naproxen (*R*/*S*): 3.45/4.16 ng/L (EW); 2.45/3.39 ng/L (SW)	Naproxen (*R*/*S*): 11.96/14.39 ng/L (EW); 8.49/11.73 ng/L (SW)	8.66–416 µg/L (*R*); 8.66–312 µg/L (*S*) EF: 0.92 (EW)	
				Ketoprofen: 0.52 ng/L (EW); 0.26/0.27 ng/L (*R*/*S*) (SW)	Ketoprofen (*R*/*S*): 1.73/1.70 ng/L (EW); 0.86/0.88 ng/L (SW)	0.83–297 µg/L (*R*); 0.83–396 µg/L (*S*) EF n.r.	
				Chloramphenicol (1*R,2R*/1S,2*S*): 2.18/2.43 ng/L (EW); 1.02/1.19 ng/L (SW)	Chloramphenicol (1*R,2R*/1S,2*S*): 7.39/8.09 ng/L (EW); 3.46/3.96 ng/L (SW)	3.40–612 µg/L (1*R,2R*); 3.33–400 µg/L (1S,2*S*) EF n.r.	
				Aminorex: 0.12 ng/L (EW); 0.06 ng/L (SW)	0.39 ng/L (EW); 0.20 ng/L (SW)	0.17–100 µg/L EF n.r.	
				Tetramisole (*R*/*S*): 1.04/0.93 ng/L (EW); 0.48/0.47 ng/L (SW)	Tetramisole (*R*/*S*): 3.42/3.08 ng/L (EW); 1.58/1.56 ng/L (SW)	1.65–396 µg/L (*R*); 1.65–297 µg/L (*S*) EF: 0.50 (EW)	
				Ifosfamide: 0.09/0.08 ng/L (E1/E2) (EW); 0.04 ng/L (SW)	Ifosfamide (E1/E2): 0.31/0.29 ng/L (EW); 0.14/0.15 ng/L (SW)	0.17–51 µg/L EF n.r.	
				3-*N*-Dechloroethy-lifosfamide (E1/E2): 3.33/2.94 ng/L (EW); 1.09 /1.14 ng/L (SW)	3-*N*-Dechloroethy-lifosfamide (E1/E2): 11.10/9.79 ng/L (EW); 3.62/3.78 ng/L (SW)	0.08–40 µg/L EF n.r.	
				Fexofenadine (E1/E2): 56.02/58.10 ng/L (EW); 33/34.66 ng/L (SW)	Fexofenadine (E1/E2): 190.29/197.33 ng/L (EW); 112.10/117.73 ng/L (SW)	136–306 µg/L (E1); 136–408 µg/L (E2) EF: 0.55 (EW)	
				10,11-Dihydro-10-hydroxy-carbama-zepine (E1/E2): 1.08/1.06 ng/L (EW); 0.53/0.54 ng/L (SW)	10,11-Dihydro-10-hydroxy-carbama-zepine (E1/E2): 3.58/3.53 ng/L (EW); 1.75/1.79 ng/L (SW)	1.67–100 µg/L (E1); 1.67–300 µg/L (E2) EF n.r.	
				Praziquantel (E1/E2): 4.54/4.83 ng/L (EW); 2.52/2.21 ng/L (SW)	Praziquantel (E1/E2): 15.12/16.07 ng/L (EW); 8.38/7.37 ng/L (SW)	8.33–400 µg/L (E1); 8.33–200 µg/L (E2) EF n.r.	
**Naproxen**	Influent wastewater (IW); Effluent wastewater (EW); River water (RW)	LC/ESI-MS/MS	Chiralpak AD-RH (150 × 4.6 mm)	n.r.	n.r.	EF: 1 (IW); 0.88–0.91 (EW); 0.84–0.98 (RW)	[41]
**Omeprazole, Lansoprazole, Rabeprazole, Pantoprazole**	Influent wastewater (IW); Effluent wastewater (EW); River water (RW)	LC/ESI-MS/MS	Chiralpak IC (250 × 4.6 mm, 5 µm)	Omeprazole: 2.03/2.29 ng/L (*R*/*S*) (IW); 0.74 ng/L (EW); 0.67/0.68 ng/L (*R*/*S*) (RW)	Omeprazole: 2.03/2.29 ng/L (*R*/*S*) (IW); 2.81 ng/L (EW); 2.55/2.59 ng/L (*R*/*S*) (RW)	2–500 µg/L EF: 0.70 (IW); 0.53 (EW); 0.54 (RW)	[43]
				Lansoprazole: 0.96/1.02 ng/L (*R*/*S*) (IW); 0.69/0.70 ng/L (*R*/*S*) (EW); 0.67 ng/L (RW)	Lansoprazole (*R*/*S*): 4.34/4.63 ng/L (IW); 3.13/3.20 ng/L (EW); 3.05/3.06 ng/L (RW)	2–500 µg/L EF: 0.51 (IW); 0.52 (EW, RW)	
				Rabeprazole: 0.94/0.95 ng/L (*R*/*S*) (IW); 0.71/0.73 ng/L (*R*/*S*) (EW); 0.78 ng/L (RW)	Rabeprazole (*R*/*S*): 3.37/3.40 ng/L (IW); 2.54/2.62 ng/L (EW); 2.81/2.78 ng/L (RW)	2–500 µg/L EF: 0.52 (IW); 0.51 (RW)	
				Pantoprazole (E1/E2): 0.96/0.94 ng/L (IW); 0.93/1 ng/L (EW); 0.96/0.91 ng/L (RW)	Pantoprazole (E1/E2): 2.99/2.94 ng/L (IW); 2.90/3.12 ng/L (EW); 2.99/2.83 ng/L (RW)	2–500 µg/L EF: 0.54 (IW); 0.51 (EW); 0.53 (RW)	
**Econazole, Miconazole, Tebuconazole, Ketoconazole**	Raw wastewater (RaW); Treated wastewater (TW); Sludge (Sd)	LC/ESI-MS/MS	AGP column (100 × 4 mm, 5 µm); AGP guard column (10 × 4 mm)	n.r.	Econazole: 0.5 ng/L (RaW); 0.3 ng/L (TW); 3 ng/g (Sd)	0.5–250 ng/mL EF: 0.50 (Sd)	[166]
					Miconazole: 0.5 ng/L (RaW); 0.3 ng/L (TW); 3 ng/g (Sd)	0.5–250 ng/mL EF: 0.5 (RaW); 0.47 (TW); 0.5 (Sd)	
					Tebuconazole: 0.8 ng/L/0.9 ng/L (E1/E2) (RaW); 0.3 ng/L (TW); 4/5 ng/g (E1/E2) (Sd)	0.5–250 ng/mL EF n.r.	
			HSA column (100 × 2 mm, 5 µm); HSA guard column (10 × 2 mm)	n.r.	Ketoconazole: 10 ng/L (RaW); 5 ng/L (TW); 29 ng/g (Sd)	5–250 ng/mL EF: 0.48 (RaW, TW, Sd)	
**Econazole, Miconazole, Tebuconazole, Ketoconazole**	River water (RW); Sludge (Sd)	LC/ESI-MS/MS	AGP column (100 × 4 mm, 5 µm); AGP guard column (10 × 4 mm)	n.r.	Econazole: 0.5 ng/L (RW); 3 ng/g (Sd)	0.5–250 ng/mL (Sd) EF: 0.52 (RW); 0.50 (Sd)	[167]
					Miconazole: 0.6 ng/L (RW); 3ng/g (Sd)	0.5–250 ng/mL (Sd) EF: 0.49–0.54 (RW); 0.50–0.52 (Sd)	
					Tebuconazole: 0.6 ng/L (RW)	EF: 0.47–0.61 (RW)	
			HSA column (100 × 2 mm, 5 µm); HSA guard column (10 × 2 mm)	n.r.	Ketoconazole: 7 ng/L (RW); 29 ng/g (Sd)	5–250 ng/mL (Sd) EF: 0.48–0.49 (Sd)	
**Tebuconazole, Hexaconazole, Penconazole, Triadimefon**	River water	LC/ESI-MS/MS	Chiralpak IC (250 × 4.6 mm, 5 µm)	Tebuconazole: 19.8 µg/L (‒); 25.4 µg/L (+)	60 µg/L (‒); 76.2 µg/L (+)	30–1500 µg/L EF n.r.	[168]
				Hexaconazole: 9.1 µg/L (‒); 8.6 µg/L (+)	27.7 µg/L (‒); 25.8 µg/L (+)		
				Penconazole: 29 µg/L (‒); 27.6 µg/L (+)	88.1 µg/L (‒); 83.8 µg/L (+)		
				Triadimefon: 8.5 µg/L	25.5 µg/L		

AM: amphetamine; *D*-citalopram: desmethyl-citalopram; EF: enantiomeric fraction; ESI: electrospray ionization; FD: fluorescence detector; FLX: fluoxetin; GC: gas chromatography; HPLC: high performance liquid chromatography; LC: liquid chromatography; LOD: limit of detection; LOQ: limit of quantification; MA: methamphetamine; MDA: 3,4-methylenedioxyamphetamine; MDL: method detection limit; MDMA: 3,4 methylenedioxymethamphetamine; MDEA: *N*-methyl-diethanolamine; MET: metoprolol; MS: mass spectrometry; MS/MS: tandem mass spectrometry; MQL: method quantification limit; NFLX: norfluoxetin; PHO: propranolol; QTOF: quadrupole time of flight mass spectrometer; OD-VNF: *O*-desmethylvenlafaxine; SBT: salbutamol; T: tramadol; UHPSFC: ultra high performance supercritical fluid chromatography; UPLC: ultra performance liquid chromatography; UV: ultraviolet detector; VNF: venlafaxine. n.r.: not referred.
